# Morphine-induced intestinal microbial dysbiosis drives TLR-dependent IgA targeting of gram-positive bacteria and upregulation of CD11b and TLR2 on a sub-population of IgA^+^ B cells

**DOI:** 10.1080/19490976.2024.2417729

**Published:** 2024-10-23

**Authors:** Nicolas Vitari, Salma Singh, Junyi Tao, Bridget Truitt, Udhghatri Kolli, Richa Jalodia, Kathryn M LaPorte, Yaa Abu, Danielle Antoine, Umakant Sharma, Sabita Roy

**Affiliations:** a Department of Microbiology and Immunology, University of Miami, Miller School of Medicine, Miami, USA; b Department of Surgery, University of Miami, Miller School of Medicine, Miami, USA; c Neuroscience Graduate Program, University of Miami, Miller School of Medicine, Miami, USA

**Keywords:** IgA, opioids, intestinal immunity, microbiome, small intestine, immunoglobulin a, IgA-seq, morphine, TLR2, IgA+CD11b+ cells

## Abstract

IgA binding dictates the composition of the intestinal microbiome and reflects dysbiotic states during chronic disease. Both pathogenic and commensal bacteria differentially bind to IgA with varying outcomes. Little is known regarding IgA dynamics immediately following microbial dysbiosis. Recent work shows that morphine treatment rapidly induces microbial dysbiosis within hours of administration. This microbial shift is characterized by the expansion of pathogenic bacteria with a concurrent decrease in commensal bacteria. Because of this rapid microbial shift, a murine model of chronic morphine treatment was used to gain insight on the host IgA response during early microbial disruption. Within 24 h, morphine treatment induces microbial dysbiosis which disrupts IgA-bacterial homeostasis, resulting in an increased concentration of unbound IgA with a corresponding decrease in the frequency of IgA-bound bacteria. Additionally, the increased concentration of unbound IgA is dependent on the microbiome, as microbial depletion abolishes the increase. At 48 h of morphine treatment, the frequency of IgA-bound bacteria increases and IgA-seq reveals increased IgA targeting of gram-positive bacteria. Both a whole-body TLR2 KO and treatment with the TLR inhibitor OxPAPC resulted in abrogation of IgA binding to bacteria, implicating modulation of IgA binding through TLR signaling. Finally, we identify that a sub-population of IgA^+^ B cells in the intestinal lamina propria has increased CD11b and TLR2 expression at 24 h of morphine treatment which could be a potential source of the observed IgA that targets gram-positive bacteria. Together, we demonstrate for the first time the role of TLR2 in IgA targeting of intestinal bacteria, and this study sheds light on the IgA dynamics during the initial hours of microbial dysbiosis.

## Introduction

The microbiome is composed of trillions of bacteria, fungi, and viruses that influence the intestinal and overall health of the host. Dysregulation of the intestinal microbiome has been linked to chronic diseases, with specific microbial pathogens dictating disease progression.^1,2^ Due to the importance of microbiome health, it is increasingly relevant to understand the interactions between the host and bacterial symbionts during disease.^3^ Intestinal immunoglobulin A (IgA) is critical in maintaining homeostasis between the immune system and gut microbiome.^4–6^ IgA is a primary mediator between the host and intestinal bacteria which can influence, and be influenced by, disease states.

Recent work highlights how context-dependent IgA binding influences the gut microbiome and overall health in multiple diseases.^7–10^ IgA binding to bacteria results in a variety of outcomes.^4,11^ IgA targets pathogenic bacteria to limit growth during disease states, but IgA binding is insufficient for clearance of the pathogenic bacteria.^2^ Inversely, high IgA binding to commensal bacteria can confer competitive advantages for survival.^12^ Loss of IgA binding can play important roles during disease.^7,13^ Importantly, recent studies illustrate the complexity of IgA-bacterial interactions, as IgA interacts with both pathogenic and commensal bacteria resulting in differing outcomes.^14^ Current efforts are aimed to understand the highly complex and ever-changing IgA-bacterial interactions in the context of chronic disease.

Opioids are the gold standard for moderate-to-severe pain management since their widespread medicinal use beginning in the 1800s.^15^ Morphine, among other opioids, is commonly prescribed and acts primarily through the Mu opioid receptor.^16^ Despite its effectiveness in pain management, morphine use is also associated with negative physiological effects, including gastrointestinal issues such as constipation, bloating, increased gut barrier disruption, and microbial dysbiosis.^17,18^ Mu opioid receptors are present at higher levels in the ileum,^19^ which makes the ileum a primary site for opioid-induced gut complications.^18,20^ Importantly, toll-like receptors (TLRs) are crucial for morphine-induced complications, and multiple recent reports have implicated TLR signaling in intestinal pathology caused by morphine treatment.^17,21–25^

TLRs detect microbial patterns and are necessary for protection against pathogenic invaders. TLRs are expressed on intestinal immune cells and epithelial cells, both contributing to intestinal defense.^26^ Notably, intestinal TLR signaling results in B cell recruitment and IgA class switching.^27^ Also, fully differentiated plasma cells constitutively express TLRs and can increase antibody production upon TLR stimulation *in vitro*.^28^ Intestinal microbiome-inducible IgA^+^CD11b^+^ plasma cells are dependent on MyD88 signaling which is an important downstream mediator of TLR signaling.^29,30^ Together, it is plausible that a subset of intestinal IgA^+^ cells that express TLRs can respond rapidly to alterations in the microbiome.

Previous work has described IgA acting on the microbiome in murine models over days to weeks of chronic inflammation^2,31^ or with long-term alteration in diet.^7,13^ However, morphine causes microbial dysbiosis along a much-accelerated timeframe, providing insights into IgA dynamics within hours of insult.^17^ Here, we illustrate that homeostasis between IgA and small intestinal bacteria is disrupted during the initial hours of morphine treatment. We then demonstrate that morphine induces IgA targeting of gram-positive bacteria, and this effect is abolished in TLR2 KO mice. Additionally, we report that TLR antagonism prevents the increased proportion of IgA-bound bacteria. Finally, we identify that a sub-population of IgA^+^ B cells in the intestinal lamina propria increase CD11b and TLR2 expression following morphine treatment. This work deconstructs the complex dynamics of intestinal IgA biology during the first hours of microbial dysbiosis. Importantly, we highlight the near immediate recognition of intestinal microbial dysbiosis that underscores the synergy between innate and adaptive immunity.

## Results

### Increased concentration of unbound IgA accompanies microbial dysbiosis within 24 h of morphine treatment

**Figure 1.**
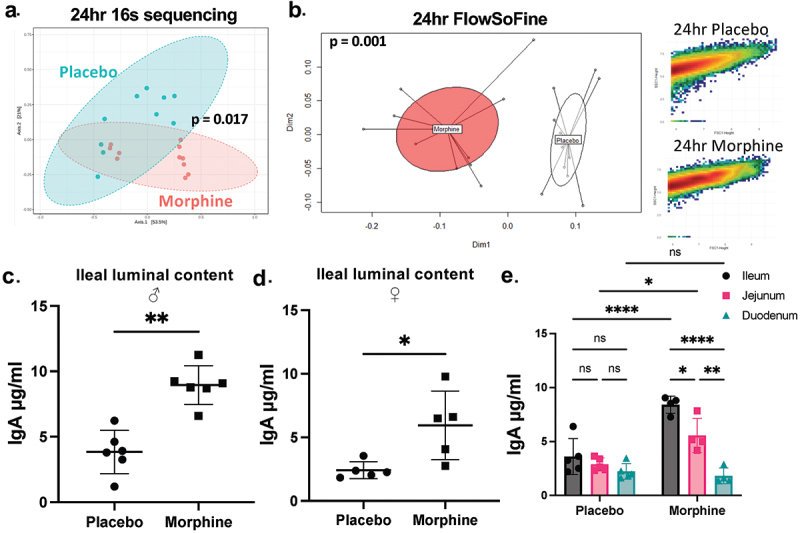
Morphine treatment induces intestinal microbial dysbiosis accompanied by increased concentration of unbound IgA. (a). 16s rRNA sequencing was performed on bacteria isolated from ileal luminal content from mice sacrificed after 24 h of continuous morphine treatment (*n* = 10-11). Principal coordinate analysis (PCoA) plots with Bray-Curtis distance were generated from 16s rRNA sequencing. (b). FlowSoFine and vegan R packages were used to generate a PCoA plot (left panel) with Bray-Curtis distance from FCS files of bacterial flow cytometry. Representative scatter plots of individual samples from each group (right panels) from FlowSoFine. (c-e). Unbound IgA concentration was measured via ELISA from luminal content taken from duodenum, jejunum, or ileum after 24 h of 25 mg morphine or placebo treatment (*n* = 4-6). Symbols represent individual mice. Data points are pooled from 2–3 independent experiments. Adjusted p-values were displayed with Benjamini-Hochberg correction method (a and b). *p < 0.05 **p < 0.01 ***p < 0.001 ****p < 0.0001 using Mann–Whitney U test (c, and d) or two-way analysis of variance with Tukey correction (e).

Consistent with previous studies, morphine treatment induces concurrent gut barrier disruption, decreased intestinal motility, and microbial dysbiosis in the ileum of mice (Fig S1A-E). While morphine-induced microbial dysbiosis has been quantified by 16s rRNA and whole-genome sequencing,^17,20,32^ recent studies show alterations in microbial composition can also be detected using flow cytometry on bacteria isolated from various sources.^33,34^ To investigate how IgA is impacted by morphine-induced microbial dysbiosis, we first studied changes in the microbiome by 16s rRNA sequencing and flow cytometry on bacteria isolated from the ileal luminal content of mice implanted with a 25 mg morphine pellet or placebo control for 24 h. Consistent with previously reported data, 16s rRNA sequencing revealed significant differences in microbial composition between morphine- and placebo-treated animals following 24 h of morphine treatment (Figure 1a). Specifically, morphine caused an increased relative abundance of gram-positive bacteria including *Enterococcus*, *Staphylococcus*, and *Parasutterella*, with a corresponding decrease in commensal bacteria such as *Lachnospiraceae* and *Turicibacter* (Fig S1C and D). Additionally, the bacterial load in the ileal luminal content increased following 24 h of morphine treatment (Fig S1F). Following flow cytometry on bacteria isolated from the ileal luminal content, we used the open-source software FlowSoFine^33^ to compare the size and granularity (FSC and SSC) of bacteria between placebo- and morphine-treated animals at 24 h. Notably, significant differences in microbial patterns were detected between treatment and control groups (Figure 1b). These data demonstrate that 24 h of morphine treatment induces microbial dysbiosis that is detected by both 16s rRNA sequencing and bacterial flow cytometry.

IgA is constitutively produced toward the intestinal microbiota and is a mediator of microbiome alterations.^11^ Since IgA plays an important role maintaining intestinal microbial homeostasis, we investigated whether the concentration of intestinal IgA is altered during morphine-induced microbial dysbiosis. Morphine increases the concentration of unbound IgA in the ileal luminal content after 24 h (Figure 1c). The increase in unbound IgA was independent of sex (Figure 1d) or strain (Fig S2A). The morphine-induced increase in IgA concentration was found to be most prominently elevated in the ileum, weakly elevated in the jejunum, and was not changed in the duodenum (Figure 1e). Additionally, the increase in unbound IgA was not observed in the cecal or large intestinal luminal contents at 24 h of morphine treatment (Fig S2B). Notably, the increased concentration of IgA was localized to the small intestine, which underscores the importance of this intestinal region in maintaining the relationship between host and microbiome. Clonidine, a non-opioid that also reduces intestinal transit time,^35^ fails to induce an increase in the concentration of unbound IgA, suggesting that reduced intestinal transit alone is not sufficient to cause the increased IgA (Fig S2C).

### Morphine-induced increase in unbound IgA concentration in the ileum is a consequence of microbial dysbiosis

**Figure 2.**
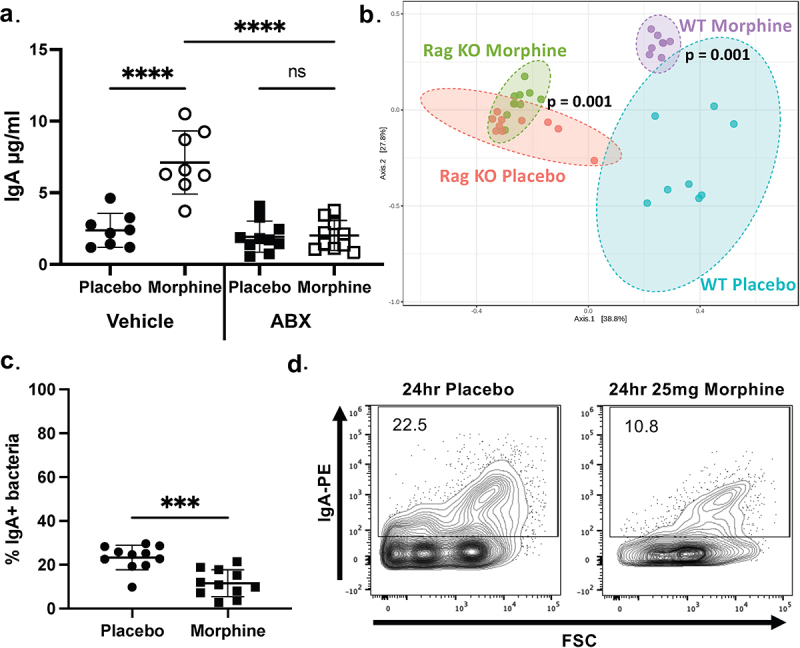
An intact microbiome is required for the morphine-induced increase in ileum IgA concentration. (a). IgA ELISA of ileal luminal content from microbiome-depleted or vehicle-treated mice following 24 h of continuous morphine treatment (*n* = 8-10). (b). PCoA plots of 16s rRNA sequencing from ileal luminal content taken from WT and RAG KO mice 24 h after implantation with 25 mg morphine or placebo pellets (*n* = 8-10). (c). Percent IgA-bound bacteria from ileal luminal content isolated from WT mice 24 h after implantation with 25 mg slow-release morphine or placebo pellet (*n* = 11). (d). Representative flow cytometry plots of DAPI^+^IgA^+^ events from (c). Symbols represent individual mice. Mean and standard deviation are shown. Data points are pooled from 3-4 independent experiments. *p < 0.05 **p < 0.01 ***p < 0.001 ****p < 0.0001 using two-way analysis of variance with Tukey correction (a), Benjamini–Hochberg correction method (b) and Mann–Whitney U test (c).

The microbiome and IgA have a bi-directional relationship – IgA can drive microbiome alterations and vice versa. The seemingly simultaneous onset of changes in both microbial composition and IgA levels raises the question of whether IgA is necessary for morphine-induced dysbiosis or if the microbial dysbiosis induces an alteration in IgA. To address this, we first sought to establish if the morphine-induced increase in intestinal IgA concentration is dependent on the microbiome. Using a pan-bacteria antibiotic cocktail, we depleted the microbiome prior to morphine treatment. Microbiome depletion prior to morphine treatment abolished the morphine-induced increase in IgA concentration compared to controls (Figure 2a). Additionally, morphine induces microbial dysbiosis within 16 h,^20^ however, the increase in unbound IgA in the ileal luminal content is not present at 16 h of morphine treatment (Fig S3A). This confirms that microbial dysbiosis precedes the alterations in IgA concentration. Together, these data suggest that the microbiome is required for the morphine-induced increase in the concentration of unbound IgA.

IgA-mediated microbial dysbiosis is still plausible since the immune system is required for morphine-induced microbial dysbiosis.^17^ Therefore, we used mice lacking the recombinase activating gene (RAG), which are deficient in B and T cells, to investigate if IgA is required for the morphine-induced microbial dysbiosis. Despite previously described baseline microbiome differences,^36^ the microbiome of morphine-treated RAG KO mice was significantly different compared to placebo-treated controls (Figure 2b). Additionally, the microbial dysbiosis observed in morphine-treated RAG KO mice was characterized by increased relative abundance of *Enterococcus*, *Staphylococcus*, and *Akkermansia* with a corresponding decrease in the commensal bacteria *Lachnospiraceae* and *Turicibacter* (Fig S3B and C) which are shown to contribute to morphine-induced dysbiosis in wild-type (WT) mice (Fig S1). Thus, IgA is dispensable for the onset of morphine-induced microbial dysbiosis, but the presence of the microbiome is required for the increased IgA concentration. Although IgA is not required for the onset of dysbiosis, IgA interactions with bacteria may be impacted by morphine treatment. Consequently, we tested whether the frequency of IgA binding to bacteria is impacted by 24 h of morphine treatment. Bacteria were isolated from the ileal luminal content and analyzed via flow cytometry (Fig S4). The percentage of IgA-bound bacteria from the ileal luminal content was significantly reduced following 24 h of morphine treatment compared to placebo-treated animals (Figure 2c,d), and the absolute number of IgA-unbound bacteria increases at 24 h of morphine treatment (Table S1). The decreased frequency of IgA-bound bacteria is in stark contrast to the increased concentration of unbound IgA detected at 24 h, suggesting that these observations may be related.

To address which bacteria have altered IgA binding at 24 h, IgA-sequencing (IgA-seq) was performed on bacteria isolated from the ileal luminal content after 24 h of continuous morphine treatment (Fig S5A). The IgA indices of the commensal bacteria *Dubosiella*, *Romboutsia*, and *Muribaculaceae* were significantly reduced following morphine treatment (Fig S5B and C). Importantly, none of the potentially pathogenic bacteria that expand with morphine treatment had IgA indices significantly increased compared to placebo, suggesting that the expanding bacteria are untargeted by IgA at 24 h (Fig S5B and C). *Muribaculaceae* and *Dubosiella* are present at comparable abundances in placebo- and morphine-treated mice (Fig S5D and E) which implies that the decrease in IgA binding to these bacteria is not driven by their relative abundance. These data indicate that morphine-induced microbial dysbiosis results in decreased IgA binding to commensals observed in homeostasis and that potentially pathogenic bacteria that increase due to morphine treatment evade a significant level of IgA binding at 24 h.

Morphine induces microbial dysbiosis throughout the intestinal tract.^37^ However, the increased concentration of unbound IgA is only observed in the small intestinal luminal content at 24 h (Figure 1e). Therefore, we investigated if the frequency of IgA-bound bacteria is disrupted in the cecal or large intestinal luminal contents at 24 h of morphine treatment. The proportion of IgA-bound bacteria in the cecal and large intestinal luminal contents at 24 h of morphine treatment were unchanged (Fig S6A and B). These data suggest that the observed changes are localized to the small intestine.

### Morphine induces an increase in the frequency of IgA-bound intestinal bacteria at 48 h

**Figure 3.**
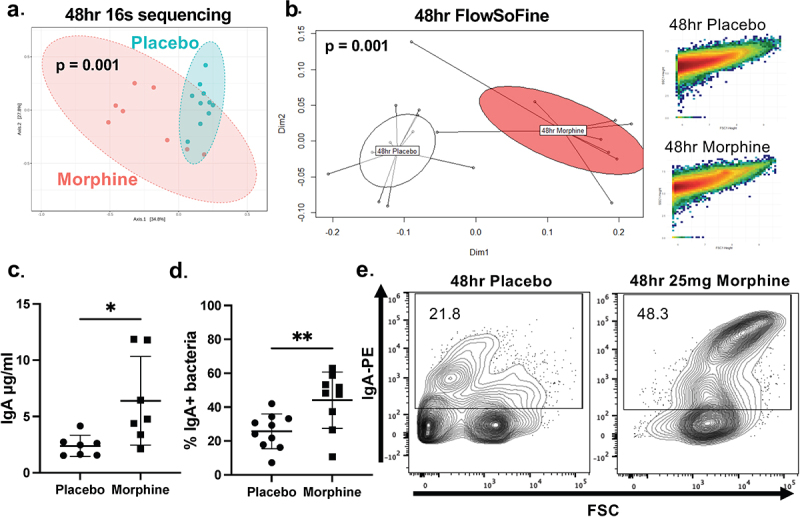
The proportion of IgA-bound intestinal bacteria increases at 48 h of morphine treatment. (a). 16s rRNA sequencing was performed on bacteria isolated from ileal luminal content from mice sacrificed after 48 h of continuous morphine treatment (*n* = 8-10). PCoA plot with Bray-Curtis distance was generated from 16s rRNA sequencing. (b). FlowSoFine and vegan R packages were used to generate a PCoA plot (left panel) with Bray-Curtis distance from FCS files of bacterial flow cytometry. Representative scatter plots of individual samples from each group (right panels) from FlowSoFine. (c). Unbound IgA concentration ELISA from ileal luminal content 48 h after implantation of 25 mg morphine or placebo pellet (*n* = 7). (d). Percent IgA-bound bacteria from ileal luminal content isolated from WT mice 48 h after implantation with 25 mg slow-release morphine or placebo pellet (*n* = 9-10). (e). Representative flow cytometry plots of DAPI^+^IgA^+^ events from (d). Symbols represent individual mice. Mean and standard deviation are shown. Data points are pooled from 2-4 independent experiments. Adjusted p-values were displayed with Benjamini–Hochberg correction method (a and b). *p < 0.05 **p < 0.01 ***p < 0.001 ****p < 0.0001 using Mann–Whitney U test (c and d).

Since morphine-induced microbial dysbiosis persists for multiple days, we sought to investigate if morphine’s impact on IgA-bacterial homeostasis would also persist. Consistent with previous work, 16s rRNA sequencing revealed that mice treated with morphine for 48 h had distinct microbial composition compared to placebo controls (Figure 3a). Importantly, this dysbiosis was characterized by an increased relative abundance of gram-positive bacteria such as *Enterococcus* and *Staphylococcus* with a concomitant decrease in commensal bacteria (Fig S7A-C). Additionally, flow cytometry analysis of bacteria isolated from the ileal luminal content confirmed that morphine-induced microbial dysbiosis continues through 48 h (Figure 3b), though the bacterial load in the ileal luminal content is not significantly different from placebo-treated mice (Fig S7D). Notably, the concentration of unbound IgA in the ileal luminal content remained elevated through 48 h (Figure 3c). Surprisingly, we observed an increase in the frequency of IgA-bound bacteria at 48 h of morphine treatment (Figure 3d,e). Together, these data demonstrate that 48 h of morphine treatment induces microbial dysbiosis which results in an increase in both the concentration of unbound IgA and the frequency of IgA-bound bacteria in the ileal luminal content.

To better understand how IgA is impacted throughout the intestine at 48 h of morphine treatment, the concentration of unbound IgA and frequency of IgA-bound bacteria were quantified in the cecal and large intestinal luminal contents. There was no alteration in the concentration of unbound IgA in the cecal or large intestinal luminal contents at 48 h of morphine treatment (Fig S8A). Consistent with these findings, there was no significant difference in the proportion of IgA-bound bacteria in the cecal or large intestinal luminal contents at 48 h of morphine treatment (Fig S8B and C).

The morphine concentration from the 25 mg slow-release pellet is highest at 24 h but reaches a stable release by 48–72 h,^38^ and morphine-induced dysbiosis persists for multiple days following implantation.^37^ Thus, we hypothesized that the alterations to IgA-bacterial homeostasis might persist. As anticipated, the composition of bacteria isolated from the ileal luminal content of morphine-treated mice was distinct from placebo-treated mice at 72 h (Fig S9A). Interestingly, the increased concentration of unbound IgA seen at both 24 and 48 h decreased by 72 h (Fig S9B). Despite the lack of increased IgA concentration, the increased proportion of IgA-bound bacteria persisted through 72 h of morphine treatment (Fig S9C and D). These data demonstrate that the morphine-induced microbial dysbiosis and increased frequency of IgA-bound bacteria continue during microbial dysbiosis and is not transient or specific to 48 h.

### Gram-positive bacteria are targeted by IgA at 48 h of morphine treatment

**Figure 4.**
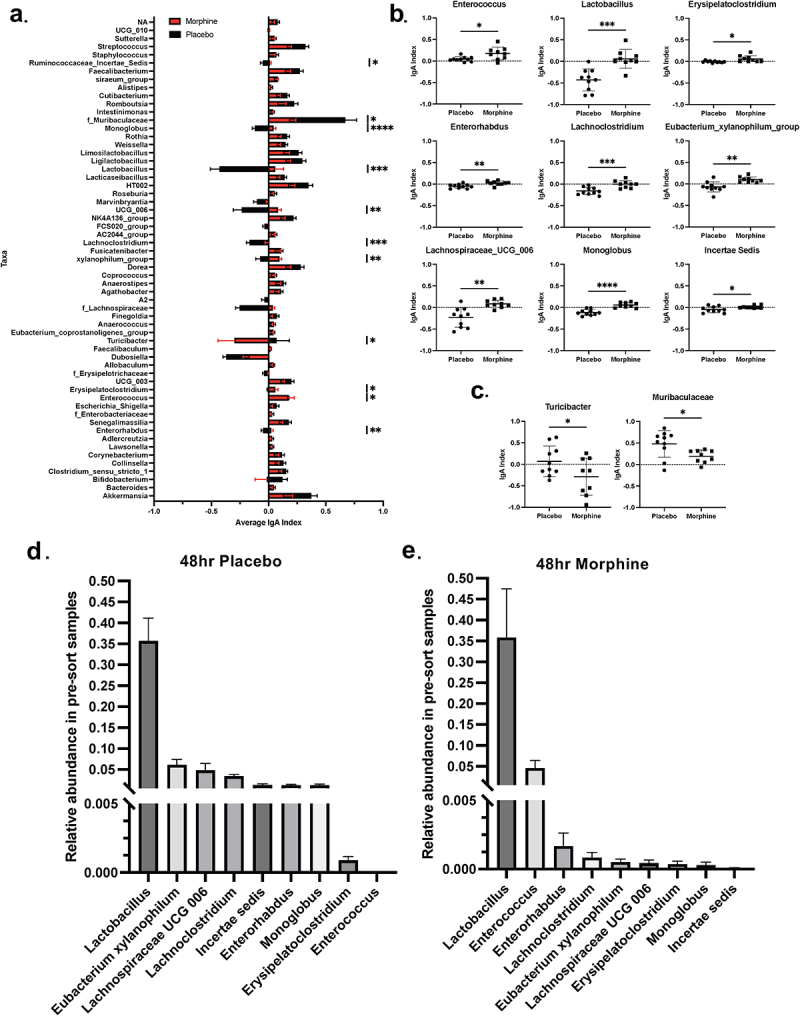
IgA binding to gram-positive bacteria is increased at 48 h of morphine treatment. (a). DNA was extracted from ileal luminal content bacteria after cell sorting to obtain IgA^+^ and IgA^−^ fractions for mice implanted with 25 mg morphine or placebo pellets for 48 h (*n* = 9-10). 16s rRNA was sequenced for all samples and IgA indices were calculated using the Kau Index.^13^ Average IgA indices for bacteria are shown (red bars = morphine, black bars = placebo). Plots showing bacterial taxa with (b) increased or (c) decreased IgA indices. Relative abundance of selected bacteria from pre-sort sequences of (d) placebo- or (e) morphine-treated mice. Symbols represent individual mice. Mean and standard deviation are shown. Data points are pooled from three independent experiments. *p < 0.05 **p < 0.01 ***p < 0.001 ****p < 0.0001 using unpaired T test (a-c).

Since the frequency of IgA binding to bacteria increases at 48 h of morphine treatment, we sought to identify which bacteria are differentially bound to IgA. IgA-seq on bacteria isolated from the ileal luminal content following 48 h of morphine or placebo treatment revealed differences in IgA binding to bacteria between placebo- and morphine-treated mice (Figure 4a). Expectedly, multiple bacterial taxa from morphine-treated mice exhibited increased IgA indices compared to placebo-treated mice (Figure 4a). The taxa with increased IgA indices were *Enterococcus*, *Enterorhabdus*, *Eubacterium xylanophilum*, *Erysipelatoclostridium*, *Lachnoclostridium, Lactobacillus*, *Monoglobus*, *Lachnospiraceae_UCG_*006, and *Incertae sedis* (Figure 4a,b). Surprisingly, all nine taxa with increased IgA indices following morphine treatment were gram-positive (Figure 4b). Of note, two commensal bacteria, *Muribaculaceae* and *Turicibacter*, had lower IgA indices following 48 h of morphine treatment (Figure 4c). It is also important to consider the relative abundance of these bacteria in the unsorted fraction. Interestingly, despite the large difference in IgA indices, *Lactobacillus* was highly abundant in both morphine and placebo groups (Figure 4d,e). Additionally, *Enterococcus* was present at a much higher relative abundance in the morphine-treated mice compared to placebo-treated controls (Figure 4d,e; Fig S7E). Taken together, these data demonstrate that ileal gram-positive bacteria are targeted by IgA at 48 h of morphine treatment.

### The morphine-induced increase in the frequency of IgA-bound bacteria is dependent on TLR2

**Figure 5.**
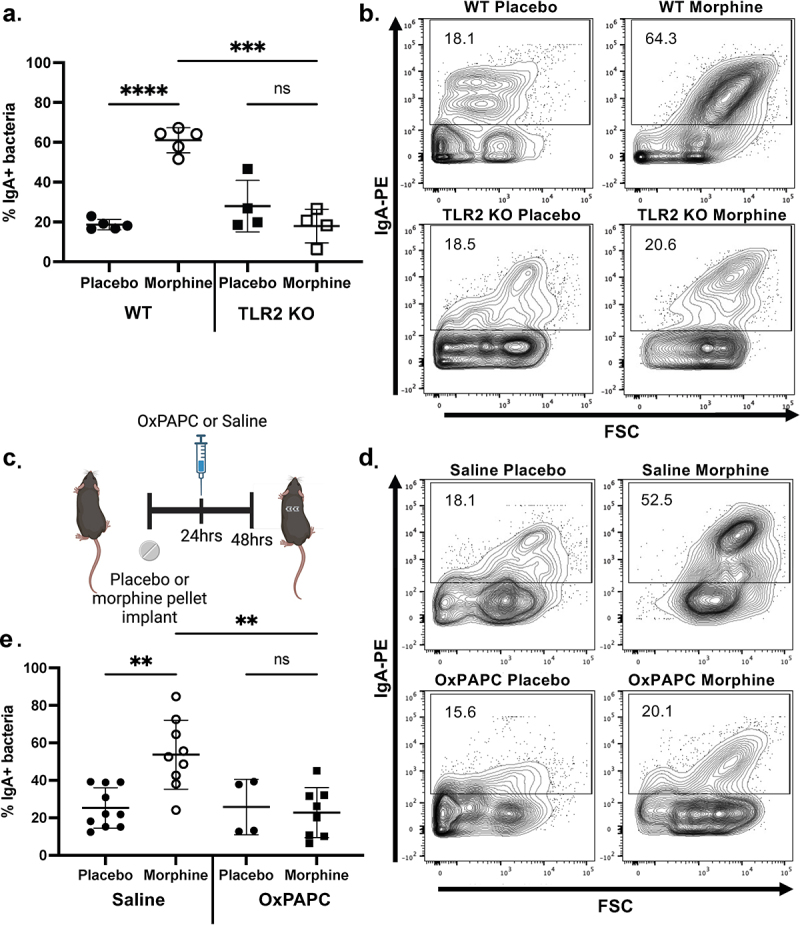
TLR2 is required for the morphine-induced increase in the frequency of IgA-bound bacteria. (a). Percent IgA-bound bacteria from ileal luminal content bacteria of WT and TLR2 KO mice implanted with 25 mg morphine or placebo pellet for 48 h (*n* = 4-5). (b). Representative flow plots of IgA-bound bacteria. (c). Mice were injected intraperitoneally with 200 μg of OxPAPC or 0.9% saline 24 h after implantation with a 25 mg morphine or placebo pellet before sacrifice at 48 h. (d). Percent IgA-bound bacteria in WT mice implanted with a 25 mg morphine or placebo pellet mice following saline or OxPAPC injection (*n* = 4-10). (e). Representative flow cytometry plots of IgA-bound bacteria. Symbols represent individual mice. Mean and standard deviation are shown. Data points are pooled from three independent experiments. *p < 0.05 **p < 0.01 ***p < 0.001 ****p < 0.0001 using two-way analysis of variance with Tukey correction (a and d).

Since the bacteria binding to IgA at 48 h of morphine treatment were gram-positive, the IgA changes during morphine treatment may be modulated by TLR2, the innate receptor that recognizes molecular patterns shared by gram-positive bacteria. To investigate if TLR2 is required for the observed increase in the percentage of IgA-bound bacteria, we implanted TLR2 KO mice with a 25 mg morphine or placebo pellet for 48 h. Morphine-treated TLR2 KO mice had a significantly lower frequency of IgA-bound bacteria than morphine-treated WT mice (Figure 5a,b). Due to the rapidity of the increased proportion of IgA-bound bacteria between 24 and 48 h, we inhibited TLR signaling at 24 h of morphine treatment. WT mice were implanted with either a placebo or morphine pellet and injected with oxidized phospholipid 1-palmitoyl-2-arachidonoyl-sn-glycero-3-phosphorylcholine (OxPAPC), a potent TLR2 and 4 inhibitor, or saline 24 h after implantation and sacrificed at 48 h (Figure 5c). Notably, OxPAPC prevented the morphine-induced increase in percent IgA binding compared to saline-treated controls (Figure 5d,e). Together, these results demonstrate that TLRs are required for the morphine-induced increase in the frequency of IgA binding to bacteria.

### Morphine causes upregulation of CD11b and TLR2 on IgA^+^ B cells in the lamina propria

**Figure 6.**
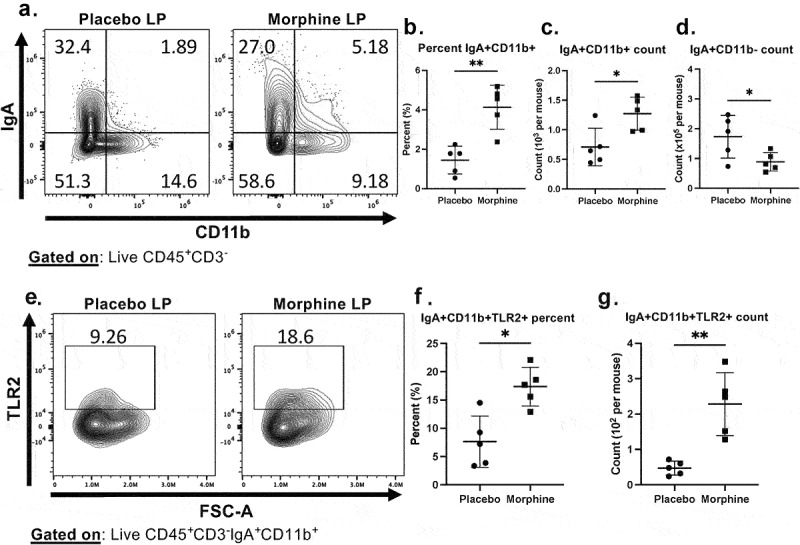
IgA^+^ immune cells in the lamina propria upregulate CD11b and TLR2 following morphine treatment (a-f). Flow cytometry on ileum lamina propria immune cells isolated 24 h after implantation with placebo or 25 mg morphine pellets (*n* = 5). (a). Representative flow cytometry plots of intestinal IgA and CD11b expression on immune cells. (b). Percentage of IgA^+^CD11b^+^ cells. (c). Absolute counts of IgA^+^CD11b^+^ cells. (d). Absolute counts of IgA^+^CD11b^−^ cells. (e) Representative flow cytometry plots of TLR2 expression on IgA^+^CD11b^+^ immune cells. (f). Percentage of IgA^+^CD11b^+^TLR2^+^ cells. (g). Absolute count of IgA^+^CD11b^+^TLR2^+^ cells. Symbols represent individual mice. Mean and standard deviation are shown. Data points are pooled from three independent experiments. *p < 0.05 **p < 0.01 ***p < 0.001 ****p < 0.0001 using two-way analysis of variance with Tukey correction (b, c, d, f, and g).

Since morphine-induced IgA targeting of gram-positive bacteria requires TLR2 signaling (Figure 5), we next investigated if TLR2 is increased on intestinal IgA^+^ B cells in the ileum lamina following morphine treatment (Fig S10). Flow cytometry and immunohistochemical analysis showed there was no significant difference in the proportion or the absolute number of IgA^+^ cells in the ileum lamina propria following 24 h of morphine treatment (Fig S11A-D). However, morphine induced an increase in the frequency and absolute number of IgA^+^CD11b^+^ cells with a corresponding decrease in the absolute number of IgA^+^CD11b^−^ cells (Figure 6a-d). These results are consistent with previous reports that TLR stimulation results in increased CD11b expression on intestinal IgA^+^ B cells.^39^ The increased CD11b expression was present on both IgA^+^B220^+^ B cells (Fig S12A and B) and IgA^+^B220^−^ plasma cells (Fig S12C and D). The increased IgA^+^CD11b^+^ cells were only observed in the ileum lamina propria and were absent in the Peyer’s Patches (Fig S12E-G). We further investigated TLR2 expression on IgA^+^CD11b^+^ cells. IgA^+^CD11b^+^TLR2^+^ cells from the lamina propria of morphine-treated were more abundant in proportion and cell number than corresponding placebo-treated controls (Figure 6e-g). These data suggest that morphine-induced microbial dysbiosis, characterized by an expansion of gram-positive bacteria, leads to upregulation of TLR2 and CD11b on lamina propria IgA^+^ B cells.

## Discussion

**Figure 7.**
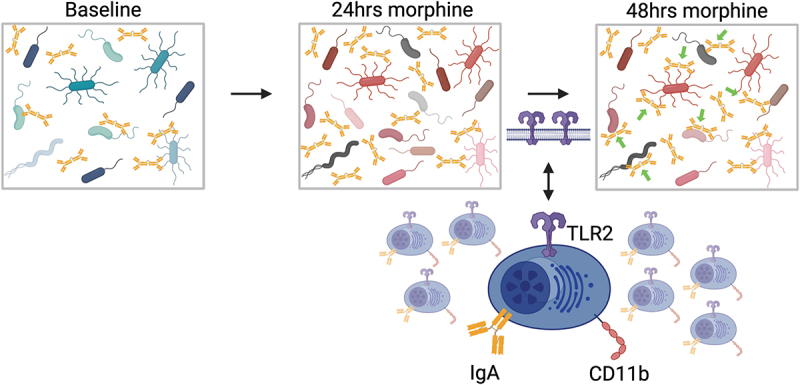
Morphine-induced microbial dysbiosis disrupts IgA-bacterial homeostasis which precedes TLR-dependent IgA targeting of intestinal bacteria.

Here, we demonstrate that morphine-induced microbial dysbiosis disrupts IgA-bacterial homeostasis in a biphasic manner, which culminates in TLR-mediated targeting of gram-positive bacteria (Figure 7). First, we report that morphine causes microbial dysbiosis which results in an increase in the concentration of unbound IgA with a corresponding decrease in the frequency of IgA-bound bacteria. Second, we report that microbial dysbiosis persists through 48 h of morphine treatment, but the frequency of IgA-bound bacteria increases while the concentration of IgA remains elevated. Next, we report that gram-positive bacteria are targeted by IgA at 48 h of morphine treatment. Further, we demonstrate that the increased frequency of IgA-bound bacteria is abolished in TLR2 whole-body KO mice and is abrogated when TLR signaling is inhibited. Finally, we demonstrate that a sub-population of IgA^+^ B cells in the intestinal lamina propria increase CD11b and TLR2 expression at 24 h of morphine treatment. Taken together, this study dissects the complex intestinal IgA-bacterial interactions which occur during the initial hours of dysbiosis and provides new evidence for the potential functional role of intestinal IgA^+^CD11b^+^ cells.

Morphine-induced microbial dysbiosis results in dynamic changes in the concentration and binding of IgA specifically in the small intestine. The site-specific change in IgA reported in our study aligns with the current understanding that IgA targeting to the microbiome originates in the ileum.^14,40^ Concurrent with morphine-induced microbial dysbiosis, gut barrier disruption and alterations in secreted metabolites in the ileum are observed.^18^ Future investigations to determine if either gut barrier disruption or secreted metabolites are important for morphine-induced changes in IgA-bacterial homeostasis would provide further insight into the mechanisms underlying IgA targeting of bacteria. Although morphine was used in this study as a Mu opioid receptor agonist that causes rapid microbial dysbiosis, future studies will investigate the effects of both synthetic and modified opioids on IgA dynamics.

Morphine treatment disrupts ileal IgA-bacterial homeostasis within 24 h. At 24 h of morphine treatment, the frequency of IgA-bound bacteria decreases, while the concentration of unbound IgA increases. Additionally, the bacterial load increases at 24 h of morphine treatment and IgA-seq reveals that expanded gram-positive bacteria are not targeted by IgA. Together, these data illustrate that expanding IgA-unbound bacteria outcompete the commensal bacteria that are bound to IgA at homeostasis, resulting in reduced frequency of bacteria bound to IgA. The IgA that is bound to commensal bacteria who are outcompeted may be released into the lumen, which could contribute to the increased concentration. In line with this, we report that some commensal bacteria have decreased IgA indices during morphine treatment. Alternatively, the host may begin to secrete IgA against the expanding bacteria, but the bacteria are not yet bound by IgA, resulting in an increased concentration of IgA in the ileal luminal content. IgA^+^ cells upregulate CD11b following morphine treatment, and IgA^+^CD11b^+^ cells are known to secrete more IgA than their CD11b^−^ counterparts.^29^ Together, these data could indicate that IgA^+^CD11b^+^ cells are starting to secrete IgA against the dysbiotic microbiome. Future studies aimed at determining the specificity of IgA secreted between 24 and 48 h of morphine treatment would shed light on the mechanism that results in the increased concentration of unbound IgA.

Morphine-induced microbial dysbiosis persists for multiple days. However, both the frequency of IgA-bound bacteria and the concentration of unbound IgA increase at 48 h of morphine treatment. The outgrowth of gram-positive bacteria present at 24 h of morphine treatment is controlled by 48 h, though the remaining bacteria are distinct from placebo-treated mice and bind to IgA in higher proportions. Increased IgA binding to multiple bacterial taxa during microbial dysbiosis is consistent with IgA binding in other disease models.^2,31^ By 48 h of morphine treatment, IgA-seq reveals an increase in IgA binding to both commensal and potentially pathogenic bacteria.^41–44^ Previous reports have noted that high IgA recognition of pathogenic bacteria reflects a state of dysbiosis and IgA binding can identify bacteria that drive pathogenesis.^2,45^ Therefore, highly IgA-bound bacteria during morphine treatment could signify which bacteria exacerbate pathology, and IgA binding could limit overgrowth of these taxa.^46^ Consistent with this idea, the alterations in the concentration of unbound IgA and IgA binding are localized to the small intestine, which could indicate that IgA limits the expanding bacteria from translocating out of the small intestine. Mono colonization experiments with potentially pathogenic or commensal gram-positive bacteria that are either free of IgA or highly bound to IgA would shed light on the functional significance of the increased frequency of IgA-bound bacteria. Although these studies provide insights into how IgA binding dynamics are impacted at 24, 48, and 72 h of morphine treatment, we are limited in drawing conclusions between time points due to differing cohorts of mice and sequencing runs. Longitudinal studies following the same cohort of mice over a period of days would provide further insight into IgA dynamics over time.

Morphine-induced intestinal pathology is exacerbated by the expansion of gram-positive bacteria,^32^ and our IgA-seq results show that IgA binding to gram-positive bacteria increases at 48 h of morphine treatment. While TLR signaling is a known contributor to morphine-mediated intestinal pathology^17,22^ and both B cells and plasma cells are known to express TLRs,^28,47^ this is the first study to connect TLR signaling with IgA targeting of bacteria during microbial dysbiosis. Although TLR stimulation in plasma cells is known to increase immunoglobulin production along a similar time frame,^28^ there is little preexisting evidence directly linking TLR signaling and IgA binding to bacteria. One link between TLRs and the microbiome are intestinal MyD88-dependent IgA^+^CD11b^+^ plasma cells which are known to proliferate and secrete IgA.^29^ We report that TLR inhibition abrogates the increased frequency of IgA binding to gram-positive bacteria during morphine treatment, and TLR2 whole-body KO mice fail to develop the increased frequency of IgA-bound bacteria. Therefore, it is plausible that TLR2-expressing IgA^+^ cells that upregulate CD11b during morphine treatment are a potential source of IgA that targets gram-positive bacteria. Consistent with previous findings, these data suggest that CD11b expression is rapidly inducible on IgA^+^ cells presumably through TLR stimulation.^39^ Further, our study is the first to link rapid microbial dysbiosis, upregulation of CD11b on IgA^+^ cells, and alterations in IgA binding to bacteria. Our study provides evidence that intestinal IgA^+^CD11b^+^ cells may play a vital role in maintaining intestinal homeostasis within hours of microbial dysbiosis. One limitation of the TLR2 whole-body KO mice is that they are resistant to morphine-induced microbial dysbiosis, which may contribute to the lack of response observed.^17,22^ To account for this limitation, TLR inhibition with OxPAPC confirms that TLRs are involved in IgA targeting of bacteria. Additionally, OxPAPC treatment reveals that TLR signaling specifically between 24 and 48 h of morphine is required for IgA targeting of gram-positive bacteria. Future studies using an inducible TLR2 KO mouse would further expand the role of TLR2 in this process.

IgA^+^CD11b^+^ cells are a potential understudied source of IgA capable of rapidly responding to microbial dysbiosis in a TLR-dependent manner. However, our findings are limited by knowledge gaps in what differentiates IgA^+^CD11b^+^ cells from other IgA^+^ immune cells. Besides proliferative ability and activation status, little is known about the signals required for cellular differentiation, and either the specificity or the function of IgA secreted by IgA^+^CD11b^+^ cells. Recent work has attributed the upregulation of CD11b on intestinal IgA^+^ immune cells through TLR signaling,^39^ and IgA^+^CD11b^+^ cells are not known to migrate to damaged intestinal sites during disease.^30^ Additional studies aimed at understanding the origin and consequences of intestinal IgA^+^CD11b^+^ cells would allow for specific targeting of these cells.

Here, we report that IgA-bacterial homeostasis is disrupted between 0 and 24 h of morphine treatment. A sub-population of IgA-secreting cells in the lamina propria rapidly upregulates CD11b and TLR2, and gram-positive bacteria are targeted by IgA in a TLR-dependent manner between 24 and 48 h of morphine treatment. IgA responses to the intestinal microbiome are essential for intestinal homeostasis, and the dynamics and complexity of intestinal IgA-bacterial interactions continue to unfold. For the first time, we demonstrate nearly immediate IgA targeting of gram-positive bacteria following rapid morphine-induced microbial dysbiosis. Understanding the host response to morphine-induced microbial dysbiosis could provide clinical benefits to help ease the microbial dysbiosis patients experience with opioid use.

## Methods

### Animals

Wild-type male and female C57BL/6, Balb/c, and RAG KO mice were purchased from Jackson Laboratories (Accession #’s: 000664, 000651, and 002216). TLR2 KO mice were purchased from Jackson Laboratories and bred in house (Accession # 022507). Prior to experimentation, mice were acclimated for at least 1 week. Mice were held in specific-pathogen-free conditions with sterile water and food provided ad libitum. All animal experiments were approved by the University of Miami Institutional Animal Care and Use Committee (IACUC). The experiments were performed in compliance with the institutional laws and guidelines.

### Morphine treatment

Twelve- to sixteen-week-old male or female mice were lightly anesthetized with isoflurane (Pivetal®) and subcutaneously implanted with a continuous release 25 mg morphine pellet or placebo pellet for 16, 24, 48, or 72 h. Morphine and placebo pellets were obtained from the National Institute on Drug Abuse. All efforts were made to minimize suffering during and after surgery.

### Clonidine treatment

Where indicated, 12-week-old WT mice were injected with 30 μg/kg clonidine or 0.9% saline twice daily for 1 day and sacrificed 24 h after first injection.

### OxPAPC treatment

OxPAPC is a potent TLR2 inhibitor that prevents both TLR1/2 and TLR2/6, as opposed to other commercially available TLR2 inhibitors.^48^ Where indicated, placebo- or morphine-treated mice were injected intraperitoneally with 200 μg of the TLR inhibitor OxPAPC (Invivogen)^49^ or 0.9% saline 24 h after implantation of 25 mg morphine or placebo pellet and sacrificed 24 h later.

### Intestinal permeability

Four hours prior to sacrifice, placebo- and morphine-treated mice were orally gavaged with 500 mg/kg 4-kDa fluorescein isothiocyanate dextran (FITC dextran) (Sigma Aldrich). Plasma was collected via cardiac puncture, and fluorescence intensity was measured using a plate reader (excitation wavelength 488 nm; emission wavelength 520 nm).

### Intestinal transit time

Intestinal transit was assessed as previously described.^35^ Briefly, placebo- or morphine-treated mice were fasted for 6 h then gavaged with 750 mg/kg 70-kDa FITC dextran or PBS. Intestinal concentrations of 70-kDa FITC dextran were determined 60 min after gavage. The small intestine was divided into three even sections: duodenum, jejunum, and ileum. Tissue and luminal contents from each section were homogenized, and large particles were removed by centrifugation (300×*g*, 3 min). Fluorescence intensity was measured using a plate reader (excitation: 485 nm, emission: 520 nm). The fluorescence signal of luminal 70-kDa FITC dextran in each segment was related to the sum of the fluorescence signals in all segments of the gastrointestinal tract.

### Microbiome depletion

The intestinal microbiome was depleted with a previously described antibiotic cocktail.^21,22^ Briefly, the mice were orally fed 0.2 ml of antibiotic cocktail or sterile water once per day for 7 days prior to the implantation of 25 mg slow-release morphine pellet. The antibiotic cocktail consisted of 10 mg/mL bacitracin, 10 mg/mL metronidazole, 40 mg/mL neomycin sulfate, 4 mg/mL vancomycin, and 24 µg/mL pimaricin dissolved in water.

### DNA extraction and 16S rRNA gene sequencing

DNA was isolated from intestinal samples using DNeasy PowerSoil Pro kit (Qiagen; catalog no. 47016). During DNA extraction, two extraction controls in each batch were included to remove potential contamination from kit reagents. Sequencing was performed by the University of Minnesota Genomics Center. The hypervariable V4 region of the 16S rRNA gene was PCR amplified using the forward primer 515F (GTGCCAFCMGCCGCGGTAA), reverse primer 806 R (GGACTACHVGGGTWTCTAAT), Illumina adaptors, and molecular barcodes to produce 427-bp amplicons. Amplicons were sequenced with the Illumina MiSeq version 3 platform, generating 300-bp paired-end reads.

### Bioinformatics analysis

Demultiplexed sequence reads were clustered into amplicon sequence variants (ASVs) with the DADA2 package (version 1.26.0)^50^ implemented in R (version 4.2.3) and RStudio (Build 353). The steps of the DADA2 pipeline include error filtering, trimming, learning of error rates, denoising, merging of paired reads, and removal of chimeras. During trimming, the forward and reverse reads were truncated at positions 230 and 190 to remove low-quality tails. Taxonomic assignment of ASVs was done at the species level using a naive Bayesian classifier^51^ implemented in DADA2 with the SILVA reference database (release 138.1).^52^ ASV and taxonomy table can be accessed from Table S2 and S3 for 24 h and 48 h samples, respectively. ASV and taxonomy tables were imported in MicrobiomeAnalyst^53^ for generating alpha and beta diversity plots, taxonomy bar plots, and linear discriminant analysis effect size (LEfSe)^54^ plots. Low count and low variance ASVs were filtered with the default threshold. Total sum scaling was used for normalization. The threshold on the logarithmic LDA score for discriminative features was set to 2. The Benjamini–Hochberg method was used for controlling the false-discovery rate (q value). The cutoff for q value was set to 0.1 for LEfSe analysis. T test was used to detect if alpha diversity differed across treatments. Permutational multivariate analysis of variance (PERMANOVA) was used to detect if beta diversity differed across treatments.

### Bacterial flow cytometry

Flow cytometry on bacteria was performed as previously described with minimal alterations.^2,13,55^ Intestinal luminal contents were squeezed out using sterile forceps, homogenized in PBS, and centrifuged at 50×g for 15 min. Supernatant was filtered through a 30um mesh, and bacteria were pelleted at 10,000×g for 10 min at 4°C. Bacterial pellets were blocked in FACS buffer (eBioscience) with 10% FBS for 20 min on ice. After washing in FACS wash buffer, bacterial samples were stained with anti-IgA-PE (1:100; eBioscience catalog no. 12-4204-82, clone mA-6E1) for 20 min at 4°C protected from light. Bacteria were washed and fixed in 2% formaldehyde for 30 min at RT protected from light. Following washing, bacteria were stained with 10 µg/ml DAPI (Thermo Fisher Scientific; catalog no. 62248) for a minimum of 3 h prior to cell sorting. Intestinal luminal content from RAG KO mice was used as a negative control.

### Microbial composition prediction with FlowSofine

Microbial dysbiosis on flow cytometry data was performed as previously described.^33^ Briefly, *R* statistical software was utilized with the FlowSoFine package on FCS files from cell sorting experiments to obtain the Bray-Curtis distance. Software available at: https://github.com/jlab/FlowSoFine.

### IgA ELISA

Unbound intestinal luminal content IgA was quantified via ELISA as previously described.^30,56,57^ Intestinal luminal contents were squeezed out using sterile forceps, weighed, and frozen at −80°C. Frozen luminal contents were diluted to 100 mg/ml in sterile PBS with 1% protease inhibitor cocktail (Invitrogen; catalog no. 78429) and vortexed for 20 min at 4°C. After centrifugation at 7000×g for 15 min, supernatant was collected and diluted 1:400 prior to analysis. Dilutions for luminal content samples were determined empirically. IgA was quantified using a Ready-Set-Go! IgA ELISA kit (Invitrogen; catalog no. 885045022) following the manufacturer’s protocol.

### Absolute bacterial quantification

Bacteria isolated from the ileal luminal content were quantified using the Invitrogen Bacteria Counting Kit according to the manufacturer’s recommendations.

### IgA-Sequencing

See Bacterial Flow Cytometry for bacterial cell sorting methods. An unsorted aliquot of each sample was collected prior to cell sorting. Bacteria from ileal luminal content samples were sorted using MoFlow Astrios EQ Cell Sorter (Beckman Coulter) on DAPI^+^IgA^+^ or DAPI^+^IgA^−^ using luminal content from RAG KO mice as negative control. A minimum of 50,000 events of each fraction were collected per sample. DNA was extracted using DNeasy PowerSoil Pro kit (Qiagen; catalog no. 47016).

### IgA indices

IgA indices were calculated using relative abundance as previously described using the Kau index.^13^ Significance between experimental groups was determined using unpaired T test.

$$\mathrm{IgA}\;\mathrm{Index}=-\left(\frac{\mathrm{Log}\left(\mathrm{RelAbun}\;\mathrm{IgA}+\right)-Log\left(\mathrm{RelAbun}\;\mathrm{IgA}-\right)}{\mathrm{Log}\left(\mathrm{RelAbun}\;\mathrm{IgA}+\right)+Log\left(\mathrm{RelAbun}\;\mathrm{IgA}-\right)}\right)$$

### Immunohistochemistry

Immunohistochemical analysis was performed as previously described.^58^ Sections from the ileum from each treatment group were fixed in 10% formalin and embedded in paraffin wax. For immunostaining, 8 μm tissue sections were deparaffinized with xylene, and the tissue was rehydrated through a series of graded alcohol and then processed for antigen retrieval using citrate antigen retrieval buffer (DAKO). Tissue sections were stained with polyclonal goat antibody against IgA (Thermo Fisher) in PBS with 1% bovine serum albumin (BSA) overnight at 4°C. After washing, sections were incubated with Alexa Fluor 488-conjugated donkey anti-goat IgG secondary antibody (Invitrogen) for 1 h at room temperature. Sections were mounted under coverslip using ProLong Gold antifade reagent with DAPI (Invitrogen). Stained sections were imaged and processed using a fluorescence microscope (Leica Microsystems, Germany).

### Immune cell flow cytometry

Immune cells from the ileum lamina propria and Peyer’s Patches were isolated using the Lamina Propria Dissociation Kit (Miltenyi Biotec catalog no. 130-097-410) according to the manufacturer’s recommendations. Isolated immune cells were washed with FACS buffer once and incubated with FC block (BD Biosciences) and Live Dead Fixable Blue dye (Invitrogen) for 15 min at 4°C. After washing twice with FACS wash, cells were stained with anti-mouse TLR2 (CB225; BD Biosciences), anti-mouse CD45 (30-F11; BD Biosciences), anti-mouse CD11b (M1/70; BD Biosciences), anti-mouse IgA (MA-6E1; Thermo Fisher Scientific), anti-mouse CD3 (145-2C11; Biolegend), and anti-mouse B220 (RA3-6B2; Biolegend). Stained cells were washed twice prior to acquisition with an Aurora Spectral Flow Cytometer (Cytek Biosciences). Data were analyzed with FlowJo.

### Statistical analysis

Prism 10.0.1 was used to perform the statistical analysis of ELISA, cytometry, intestinal permeability, and IgA index data. Details on the analyses done for each plot can be found in the corresponding figure legend. Symbols represent individual mice. Mean and standard deviation are shown. Stars denote the following p-values: *p < 0.05 **p < 0.01 ***p < 0.001 ****p < 0.0001.

## Supplementary Material

Supplemental Material

## Data Availability

Sequence data are available at the Biostudies database^59^ (https://www.ebi.ac.uk/biostudies/) under accession number (S-BSST1193).

## References

[1] Durack J, Lynch SV. The gut microbiome: relationships with disease and opportunities for therapy. J Exp Med. 2019;216(1):20–18. doi:10.1084/jem.20180448. 30322864 PMC6314516

[2] Palm NW, de Zoete M, Cullen T, Barry N, Stefanowski J, Hao L, Degnan P, Hu J, Peter I, Zhang W, et al. Immunoglobulin a coating identifies colitogenic bacteria in inflammatory bowel disease. Cell. 2014;158(5):1000–1010. doi:10.1016/j.cell.2014.08.006. 25171403 PMC4174347

[3] Zheng D, Liwinski T, Elinav E. Interaction between microbiota and immunity in health and disease. Cell Res. 2020;30(6):492–506. doi:10.1038/s41422-020-0332-7. 32433595 PMC7264227

[4] Bunker JJ, Bendelac A. IgA responses to microbiota. Immunity. 2018;49(2):211–224. doi:10.1016/j.immuni.2018.08.011. 30134201 PMC6107312

[5] Huus KE, Petersen C, Finlay BB. Diversity and dynamism of IgA-microbiota interactions. Nat Rev Immunol. 2021;21(8):514–525. doi:10.1038/s41577-021-00506-1. 33568782

[6] Weis AM, Round JL. Microbiota-antibody interactions that regulate gut homeostasis. Cell Host & Microbe. 2021;29(3):334–346. doi:10.1016/j.chom.2021.02.009. 33705705 PMC7990058

[7] Huus KE, Bauer KC, Brown EM, Bozorgmehr T, Woodward SE, Serapio-Palacios A, Boutin RCT, Petersen C, Finlay BB. Commensal bacteria modulate immunoglobulin a binding in response to host nutrition. Cell Host Microbe. 2020;27(6):909–921.e5. doi:10.1016/j.chom.2020.03.012. 32289261

[8] Donaldson GP, Ladinsky MS, Yu KB, Sanders JG, Yoo BB, Chou W-C, Conner ME, Earl AM, Knight R, Bjorkman PJ, et al. Gut microbiota utilize immunoglobulin a for mucosal colonization. Science. 2018;360(6390):795–800. doi:10.1126/science.aaq0926. 29724905 PMC5973787

[9] Moor K, Diard M, Sellin ME, Felmy B, Wotzka SY, Toska A, Bakkeren E, Arnoldini M, Bansept F, Co AD, et al. High-avidity IgA protects the intestine by enchaining growing bacteria. Nature. 2017;544(7651):498–502. doi:10.1038/nature22058. 28405025

[10] McLoughlin K, Schluter J, Rakoff-Nahoum S, Smith A, Foster K. Host selection of microbiota via differential adhesion. Cell Host & Microbe. 2016;19(4):550–559. doi:10.1016/j.chom.2016.02.021. 27053168

[11] Yang Y, Palm N. Immunoglobulin a and the microbiome. Curr Opin In Microbiol. 2020;56:89–96. doi:10.1016/j.mib.2020.08.003. 32889295

[12] Nakajima A, Vogelzang A, Maruya M, Miyajima M, Murata M, Son A, Kuwahara T, Tsuruyama T, Yamada S, Matsuura M, et al. IgA regulates the composition and metabolic function of gut microbiota by promoting symbiosis between bacteria. J Exp Med. 2018;215(8):2019–2034. doi:10.1084/jem.20180427. 30042191 PMC6080902

[13] Kau AL, Liu PJ, Rao J, Yatsunenko S, Trehan T, Manary I, Liu MJ, Stappenbeck TC, Maleta TS, Ashorn KM, et al. Functional characterization of IgA-targeted bacterial taxa from undernourished Malawian children that produce diet-dependent enteropathy. Sci Transl Med. 2015;7(276).276. doi:10.1126/scitranslmed.aaa4877. PMC4423598 25717097

[14] Pabst O, Slack E. IgA and the intestinal microbiota: the importance of being specific. Mucosal Immunol. 2020;13(1):12–21. doi:10.1038/s41385-019-0227-4. 31740744 PMC6914667

[15] Rosenblum A, Marsch LA, Joseph H, Portenoy RK. Opioids and the treatment of chronic pain: controversies, current status, and future directions. Exp Clin Psychopharmacol. 2008;16(5):405–416. doi:10.1037/a0013628. 18837637 PMC2711509

[16] Contet C, Kieffer BL, Befort K. Mu opioid receptor: a gateway to drug addiction. Curr Opin Neurobiol. 2004;14(3):370–378. doi:10.1016/j.conb.2004.05.005. 15194118

[17] Banerjee S, Sindberg G, Wang F, Meng J, Sharma U, Zhang L, Dauer P, Chen C, Dalluge J, Johnson T, et al. Opioid-induced gut microbial disruption and bile dysregulation leads to gut barrier compromise and sustained systemic inflammation. Mucosal Immunol. 2016;9(6):1418–1428. doi:10.1038/mi.2016.9. 26906406 PMC4996771

[18] Meng J, Yu H, Ma J, Wang J, Banerjee S, Charboneau R, Barke RA, Roy S. Morphine induces bacterial translocation in mice by compromising intestinal barrier function in a tlr-dependent manner. PLOS ONE. 2013;8(1):e54040. doi:10.1371/journal.pone.0054040. 23349783 PMC3548814

[19] Ross GR, Gabra BH, Dewey WL, Akbarali HI. Morphine tolerance in the mouse ileum and colon. J Pharmacol Exp Ther. 2008;327(2):561–572. doi:10.1124/jpet.108.143438. 18682567 PMC2574683

[20] Kolli U, Jalodia R, Moidunny S, Singh PK, Ban Y, Tao J, Cantu GN, Valdes E, Ramakrishnan S, Roy S, et al. Multi-omics analysis revealing the interplay between gut microbiome and the host following opioid use. Gut Microbes. 2023;15(2):2246184. doi:10.1080/19490976.2023.2246184. 37610102 PMC10448978

[21] Truitt B, Venigalla G, Singh P, Singh S, Tao J, Chupikova I, Roy S. The gut microbiome contributes to somatic morphine withdrawal behavior and implicates a TLR2 mediated mechanism. Gut Microbes. 2023;15(1):2242610. doi:10.1080/19490976.2023.2242610. 37589387 PMC10438851

[22] Zhang L, Meng J, Ban Y, Jalodia R, Chupikova I, Fernandez I, Brito N, Sharma U, Abreu MT, Ramakrishnan S, et al. Morphine tolerance is attenuated in germfree mice and reversed by probiotics, implicating the role of gut microbiome. Proc Natl Acad Sci USA. 2019;116(27):13523–13532. doi:10.1073/pnas.1901182116. 31209039 PMC6613141

[23] Shah M, Choi S. Toll-like receptor-dependent negative effects of opioids: a battle between analgesia and Hyperalgesia. Front Immunol. 2017;8:642. doi:10.3389/fimmu.2017.00642. 28620391 PMC5450035

[24] Eisenstein TK. The role of opioid receptors in immune system function. Front Immunol. 2019;10:2904. doi:10.3389/fimmu.2019.02904. 31921165 PMC6934131

[25] Plein LM, Rittner HL. Opioids and the immune system – friend or foe. Br J Pharmacol. 2018;175(14):2717–2725. doi:10.1111/bph.13750. 28213891 PMC6016673

[26] Price AE, Shamardani K, Lugo KA, Deguine J, Roberts AW, Lee BL, Barton GM. A map of Toll-like receptor expression in the intestinal epithelium reveals distinct spatial, cell type-specific, and temporal patterns. Immunity. 2018;49(3):560–575.e6. doi:10.1016/j.immuni.2018.07.016. 30170812 PMC6152941

[27] Shang L, Fukata M, Thirunarayanan N, Martin AP, Arnaboldi P, Maussang D, Berin C, Unkeless JC, Mayer L, Abreu MT, et al. Toll-like receptor signaling in small intestinal epithelium promotes B-cell recruitment and IgA production in lamina propria. Gastroenterology. 2008;135(2):529–538. doi:10.1053/j.gastro.2008.04.020. 18522803 PMC2598776

[28] Dorner M, Brandt S, Tinguely M, Zucol F, Bourquin JP, Zauner L, Berger C, Bernasconi M, Speck RF, Nadal D. Plasma cell toll‐like receptor (TLR) expression differs from that of B cells, and plasma cell TLR triggering enhances immunoglobulin production. Immunology. 2009;128(4):573–579. doi:10.1111/j.1365-2567.2009.03143.x. 19950420 PMC2792141

[29] Kunisawa J, Gohda M, Hashimoto E, Ishikawa I, Higuchi M, Suzuki Y, Goto Y, Panea C, Ivanov II, Sumiya R, et al. Microbe-dependent CD11b+ IgA+ plasma cells mediate robust early-phase intestinal IgA responses in mice. Nat Commun. 2013;4(1):1772. doi:10.1038/ncomms2718. 23612313 PMC3644083

[30] Fu Y, Wang Z, Yu B, Lin Y, Huang E, Liu R, Zhao C, Lu M, Xu W, Liu H, et al. Intestinal CD11b(+) B cells ameliorate colitis by secreting immunoglobulin a. Front Immunol. 2021;12:697725. doi:10.3389/fimmu.2021.697725. 34804004 PMC8595478

[31] Shapiro JM, de Zoete MR, Palm NW, Laenen Y, Bright R, Mallette M, Bu K, Bielecka AA, Xu F, Hurtado-Lorenzo A, et al. Immunoglobulin a targets a unique subset of the microbiota in inflammatory bowel disease. Cell Host Microbe. 2021;29(1):83–93 e3. doi:10.1016/j.chom.2020.12.003. 33385335 PMC10477929

[32] Meng J, Banerjee S, Li D, Sindberg GM, Wang F, Ma J, Roy S. Opioid exacerbation of gram-positive sepsis, induced by gut microbial modulation, is rescued by IL-17A neutralization. Sci Rep. 2015;5(1):5. doi:10.1038/srep10918. PMC4454150 26039416

[33] Kupschus J, Janssen S, Hoek A, Kuska J, Rathjens J, Sonntag C, Ickstadt K, Budzinski L, Chang H-D, Rossi A, et al. Rapid detection and online analysis of microbial changes through flow cytometry. Cytometry A. 2022;103(5):419–428. doi:10.1002/cyto.a.24704. 36354152

[34] Koch C, Günther S, Desta AF, Hübschmann T, Müller S. Cytometric fingerprinting for analyzing microbial intracommunity structure variation and identifying subcommunity function. Nat Protoc. 2013;8(1):190–202. doi:10.1038/nprot.2012.149. 23288319

[35] Meng J, Abu YF, Zhang Y, Zhou Y, Xie Y, Yan Y, Tao J, Ramakrishnan S, Chen C, Roy S, et al. Opioid-induced microbial dysbiosis disrupts irinotecan (CPT-11) metabolism and increases gastrointestinal toxicity in a murine model. Br J Pharmacol. 2023;180(10):1362–1378. doi:10.1111/bph.16020. 36562107 PMC10089971

[36] Zhang H, Sparks JB, Karyala SV, Settlage R, Luo XM. Host adaptive immunity alters gut microbiota. Isme J. 2015;9(3):770–781. doi:10.1038/ismej.2014.165. 25216087 PMC4331585

[37] Wang F, Meng J, Zhang L, Johnson T, Chen C, Roy S. Morphine induces changes in the gut microbiome and metabolome in a morphine dependence model. Sci Rep. 2018;8(1):3596. doi:10.1038/s41598-018-21915-8. 29483538 PMC5827657

[38] McLane VD, Bergquist I, Cormier J, Barlow DJ, Houseknecht KL, Bilsky EJ, Cao L. Long-term morphine delivery via slow release morphine pellets or osmotic pumps: plasma concentration, analgesia, and naloxone-precipitated withdrawal. Life Sci. 2017;185:1–7. doi:10.1016/j.lfs.2017.07.016. 28723417 PMC5577921

[39] Gao P, Adachi T, Okai S, Morita N, Kitamura D, Shinkura R. Integrin CD11b provides a new marker of pre-germinal center IgA+ B cells in murine Peyer’s patches. Int Immunol. 2022;34(5):249–262. doi:10.1093/intimm/dxab113. 34971392 PMC9020567

[40] Bunker JJ, Flynn T, Koval J, Shaw D, Meisel M, McDonald B, Ishizuka I, Dent A, Wilson P, Jabri B, et al. Innate and adaptive humoral responses coat distinct commensal bacteria with immunoglobulin a. Immunity. 2015;43(3):541–553. doi:10.1016/j.immuni.2015.08.007. 26320660 PMC4575282

[41] Clavel T, Duck W, Charrier C, Wenning M, Elson C, Haller D. 2009. Int J Syst Evol Microbiol. 2010;60(Pt 7):1527–1531. doi:10.1099/ijs.0.015016-0. 19684311 PMC3052451

[42] Li H, Wang Y, Shao S, Yu H, Wang D, Li C, Yuan Q, Liu W, Cao J, Wang X, et al. Alleviates dextran sulfate sodium salt-induced colitis in mice through anti-inflammation, regulating Th17/Treg balance, maintaining intestinal barrier integrity, and modulating gut microbiota. J Pharm Anal. 2022;12(6):824–838. doi:10.1016/j.jpha.2022.08.001. 36605573 PMC9805946

[43] Zhao X, Liu H, Wu Y, Hu N, Lei M, Zhang Y, Wang S. Intervention with the crude polysaccharides of physalis pubescens L. mitigates colitis by preventing oxidative damage, aberrant immune responses, and dysbacteriosis. J Food Sci. 2020;85(8):2596–2607. doi:10.1111/1750-3841.15330. 32696986

[44] Cai YY, Huang F-Q, Lao X, Lu Y, Gao X, Alolga RN, Yin K, Zhou X, Wang Y, Liu B, et al. Integrated metagenomics identifies a crucial role for trimethylamine-producing lachnoclostridium in promoting atherosclerosis. NPJ Biofilms Microbiomes. 2022;8(1):11. doi:10.1038/s41522-022-00273-4. 35273169 PMC8913745

[45] van der Waaij LA, Kroese FG, Visser A, Nelis GF, Westerveld BD, Jansen PL, Hunter JO. Immunoglobulin coating of faecal bacteria in inflammatory bowel disease. Eur J Gastroenterol Hepatol. 2004;16(7):669–674. doi:10.1097/01.meg.0000108346.41221.19. 15201580

[46] Suzuki K, Meek B, Doi Y, Muramatsu M, Chiba T, Honjo T, Fagarasan S. Aberrant expansion of segmented filamentous bacteria in IgA-deficient gut. Proc Natl Acad Sci U S A. 2004;101(7):1981–1986. doi:10.1073/pnas.0307317101. 14766966 PMC357038

[47] Browne EP. Regulation of B-cell responses by Toll-like receptors. Immunology. 2012;136(4):370–379. doi:10.1111/j.1365-2567.2012.03587.x. 22444240 PMC3401975

[48] Zheng M, Karki R, Williams EP, Yang D, Fitzpatrick E, Vogel P, Jonsson CB, Kanneganti T-D. TLR2 senses the SARS-CoV-2 envelope protein to produce inflammatory cytokines. Nat Immunol. 2021;22(7):829–838. doi:10.1038/s41590-021-00937-x. 33963333 PMC8882317

[49] Oskolkova OV, Afonyushkin T, Preinerstorfer B, Bicker W, von Schlieffen E, Hainzl E, Demyanets S, Schabbauer G, Lindner W, Tselepis AD, et al. Oxidized phospholipids are more potent antagonists of lipopolysaccharide than inducers of inflammation. J Immunol. 2010;185(12):7706–7712. doi:10.4049/jimmunol.0903594. 21068406

[50] Callahan BJ, McMurdie PJ, Rosen MJ, Han AW, Johnson AJA, Holmes SP. DADA2: High-resolution sample inference from Illumina amplicon data. Nat Methods. 2016;13(7):581–583. doi:10.1038/nmeth.3869. 27214047 PMC4927377

[51] Wang Q, Garrity GM, Tiedje JM, Cole JR. NaÏve bayesian Classifier for rapid assignment of rRNA sequences into the New bacterial taxonomy. Appl And Environ Microbiol. 2007;73(16):5261–5267. doi:10.1128/AEM.00062-07. 17586664 PMC1950982

[52] Yilmaz P, Parfrey LW, Yarza P, Gerken J, Pruesse E, Quast C, Schweer T, Peplies J, Ludwig W, Glöckner FO, et al. The SILVA and “all-species living tree project (LTP)” taxonomic frameworks. Nucleic Acids Res. 2014;42(D1):D643–D648. doi:10.1093/nar/gkt1209. 24293649 PMC3965112

[53] Chong J, Liu P, Zhou G, Xia J. Using MicrobiomeAnalyst for comprehensive statistical, functional, and meta-analysis of microbiome data. Nat Protoc. 2020;15(3):799–821. doi:10.1038/s41596-019-0264-1. 31942082

[54] Segata N, Izard J, Waldron L, Gevers D, Miropolsky L, Garrett WS, Huttenhower C. Metagenomic biomarker discovery and explanation. Genome Biol. 2011;12(6):R60. doi:10.1186/gb-2011-12-6-r60. 21702898 PMC3218848

[55] Jackson MA, Pearson C, Ilott NE, Huus KE, Hegazy AN, Webber J, Finlay BB, Macpherson AJ, Powrie F, Lam LH, et al. Accurate identification and quantification of commensal microbiota bound by host immunoglobulins. Microbiome. 2021;9(1):33. doi:10.1186/s40168-020-00992-w. 33516266 PMC7847592

[56] Smeekens JM, Johnson-Weaver BT, Hinton AL, Azcarate-Peril MA, Moran TP, Immormino RM, Kesselring JR, Steinbach EC, Orgel KA, Staats HF, et al. Fecal IgA, antigen absorption, and gut microbiome composition are associated with food antigen sensitization in genetically susceptible mice. Front Immunol. 2020;11:599637. doi:10.3389/fimmu.2020.599637. 33542716 PMC7850988

[57] Gai W, Zou W, Lei L, Luo J, Tu H, Zhang Y, Wang K, Tien P, Yan H. Effects of different immunization protocols and adjuvant on antibody responses to inactivated SARS-CoV vaccine. Viral Immunol. 2008;21(1):27–37. doi:10.1089/vim.2007.0079. 18355120

[58] Jalodia R, Kolli U, Braniff RG, Tao J, Abu YF, Chupikova I, Moidunny S, Ramakrishnan S, Roy S. Morphine mediated neutrophil infiltration in intestinal tissue play essential role in histological damage and microbial dysbiosis. Gut Microbes. 2022;14(1):2143225. doi:10.1080/19490976.2022.2143225. 36409161 PMC9683065

[59] Sarkans U, Gostev M, Athar A, Behrangi E, Melnichuk O, Ali A, Minguet J, Rada JC, Snow C, Tikhonov A, et al. The BioStudies database—one stop shop for all data supporting a life sciences study. Nucleic Acids Res. 2018;46(D1):D1266–D1270. doi:10.1093/nar/gkx965. 29069414 PMC5753238

