# Supplementary material for: Morphine-induced intestinal microbial dysbiosis drives TLR-dependent IgA targeting of gram-positive bacteria and upregulation of CD11b and TLR2 on a sub-population of IgA+ B cells
Source: Gut Microbes. 2024 Oct 23;16(1):2417729. doi: 10.1080/19490976.2024.2417729 (PMC11508942; doi:10.1080/19490976.2024.2417729)
Supplement: Supplemental Material [file KGMI_A_2417729_SM8266.zip › KGMI_A_2417729/suppl_data/Vitari_GutMicrobes_9_9_24_Supplementalfigures.docx]

**Figure S1. Morphine-induced microbial dysbiosis in ileal luminal content at 24 hrs via 16s rRNA sequencing.**

**A.** Plasma FITC-dextran 24 hrs following implantation of 25 mg morphine or placebo control (n=5). **B**. Alpha diversity with Shannon index (n=10-11). **C**. LEfSe plot. **D**. Relative abundance bar plot at the genus level. **E**. Percent of 70kDa FITC-Dextran present in the small intestine compartments 24 hrs after implantation with 25 mg morphine or placebo pellet (n = 5). **F**. Absolute quantification of bacteria in the ileal luminal content 24 hrs after implantation with placebo or 25 mg morphine pellet (n = 4-5). Symbols represent individual mice. Mean and standard deviation are shown. Data points are pooled from 2-3 independent experiments. * p < 0.05 ** p < 0.01 *** p < 0.001 **** p < 0.0001 using Unpaired T test (**A** and **B**), LDA scores (**C**), Two-way Analysis of Variance (**E**) or Mann-Whitney U test (**F**).

**Figure S2.** **Morphine-induced increase in concentration of unbound IgA is not strain-specific and is limited to the small intestine.**

**A**. IgA ELISA on ileal luminal content collected from Balb/c mice after 24 hrs of 25 mg morphine or placebo pellet (n = 10-11). **B**. IgA ELISA on cecal and large intestinal luminal content collected from C57Bl/6 mice after 24 hrs of 25 mg morphine or placebo pellet (n = 5). **C**. IgA ELISA on ileal luminal content following clonidine or saline treatment (n = 5). Data points are pooled from 2-4 independent experiments. * p < 0.05 ** p < 0.01 *** p < 0.001 **** p < 0.0001 using Mann Whitney U test (**A**-**C**).

**Figure S3.** **Morphine induces microbial dysbiosis in RAG KO mice.**

**A.** IgA ELISA on ileal luminal content collected from WT mice after 16 hrs of 25 mg morphine or placebo pellet (n = 5). **B**. Alpha diversity with Shannon index of ileal luminal content of RAG KO mice 24 hrs post-implant with placebo or 25 mg morphine pellet (n = 10-11). **C**. LEfSe plot of RAG KO mice at 24 hrs of morphine treatment (n = 10). Symbols represent individual mice. Data points are pooled from 2-3 independent experiments.

**Figure S4. Gating strategy for identifying IgA bound bacteria.**

**Figure S5. Commensal bacteria have decreased IgA binding and expanding bacteria are not targeted following 24 hrs of morphine treatment.** **A**. IgA-sequencing workflow. **B**. DNA was extracted from ileal luminal content bacteria after cell sorting to obtain IgA^+^ and IgA^-^ fractions for mice implanted with 25 mg morphine or placebo pellets for 24 hrs (n = 11). 16s rRNA was sequenced for all samples and IgA indices were calculated using the Kau Index. Average IgA indices for bacteria are shown (red bars = morphine, black bars = placebo). **C**. Plots showing IgA indices for individual bacterial taxa. Relative abundance of selected bacteria from pre-sort sequences of **D**. placebo- or **E**. morphine-treated mice. Symbols represent individual mice. Mean and standard deviation are shown. Data points are pooled from 3 independent experiments. * p < 0.05 ** p < 0.01 *** p < 0.001 **** p < 0.0001 using Unpaired T Test (**B** and **C**).

**Figure S6. Frequency of IgA bound bacteria in cecal and large intestinal luminal contents are unchanged at 24 hrs of morphine treatment. A**. Percent IgA bound bacteria from cecal luminal content isolated from WT mice 24 hrs after implantation with 25 mg slow-release morphine or placebo pellet (n = 5). **B**. Percent IgA bound bacteria from large intestinal luminal content isolated from WT mice 24 hrs after implantation with 25 mg slow-release morphine or placebo pellet (n = 5). Symbols represent individual mice. Mean and standard deviation are shown. Data points are pooled from 3 independent experiments. * p < 0.05 ** p < 0.01 *** p < 0.001 **** p < 0.0001 using Mann-Whitney U test (**A** and **B**).

**Figure S7. Morphine induces microbial dysbiosis in ileal luminal content of WT mice at 48 hrs. A.** LEfSe plot (n = 8-10). **B**. Alpha diversity with Shannon index. **C**. Relative abundance bar plot at the genus level. **D**. Absolute quantification of bacteria in the ileal luminal content 48 hrs after implantation with placebo or 25 mg morphine pellet (n = 4-5). Symbols represent individual mice. Mean and standard deviation are shown. Data points are pooled from 2-3 independent experiments. * p < 0.05 ** p < 0.01 *** p < 0.001 **** p < 0.0001 using Unpaired T test (**B**), LDA scores (**C**) or Mann-Whitney U test (**D**).

**Figure S8. Frequency of IgA bound bacteria in cecal and large intestinal luminal contents are unchanged at 48 hrs of morphine treatment. A.** IgA ELISA on cecal and large intestinal luminal content collected mice 48 hrs after implantation of 25 mg morphine or placebo pellet (n = 5). **B**. Percent IgA bound bacteria from cecal luminal content isolated from WT mice 48 hrs after implantation with 25 mg slow-release morphine or placebo pellet (n = 5). **C**. Percent IgA bound bacteria from large intestinal luminal content isolated from WT mice 48 hrs after implantation with 25 mg slow-release morphine or placebo pellet (n = 5). Symbols represent individual mice. Mean and standard deviation are shown. Data points are pooled from 2 independent experiments. * p < 0.05 ** p < 0.01 *** p < 0.001 **** p < 0.0001 using Two Way Analysis of Variance with Tukey correction (**A**) and Mann-Whitney U test (**B** and **C**).

**Figure S9. Morphine-induced increase in the frequency of IgA bound bacteria persists through 72 hrs.**

**A**. FlowSoFine and vegan R packages were used to generate a PCoA plot (left panel) with Bray-Curtis distance from FCS files of bacterial flow cytometry isolated from ileal luminal content following 72 hrs of morphine treatment. Representative scatter plots of individual samples from each group (right panels) from FlowSoFine. **B**. Unbound IgA concentration ELISA from ileal luminal content 72 hrs after implantation of 25 mg morphine or placebo treatment (n = 5). **C**. Representative flow cytometry plots of DAPI^+^IgA^+^ events from ileal luminal content isolated from WT mice 72 hrs after implantation with 25 mg slow-release morphine or placebo pellet (n = 5). **D**. Summary data of **C**. Symbols represent individual mice. Symbols represent individual mice. Mean and standard deviation are shown. Data points are pooled from 2 independent experiments. Adjusted p-values were displayed with Benjamini-Hochberg correction method (**A**). * p < 0.05 ** p < 0.01 *** p < 0.001 **** p < 0.0001 using Mann Whitney U test (**B** and **D**).

**Figure S10. Gating strategy to identify intestinal IgA+ plasma and B cells isolated from the ileum.**

**Figure S11. The absolute number of lamina propria IgA^+^ cells is unchanged during morphine treatment. A-C.** Flow cytometry on ileum lamina immune cells isolated 24 hrs after implantation with placebo or 25 mg morphine pellets (n = 5)**. A.** Representative flow cytometry plots of IgA expression on CD45^+^CD3^-^ cells. **B.** Percentage of IgA^+^ immune cells. **C.** Number of IgA^+^ immune cells. **D.** Immunohistochemical analysis of IgA^+^ cells in the ileum lamina propria 24 hrs post-implantation of 25 mg morphine or placebo pellet (n = 4). Mean and standard deviation are shown. Data points are pooled from 2-3 independent experiments. * p < 0.05 ** p < 0.01 *** p < 0.001 **** p < 0.0001 using Mann-Whitney U Test **(B, C,** and **D).**

**Figure S12. Both lamina propria B220^+^IgA^+^ B cells and B220^+^IgA^-^ plasma cells have increased CD11b expression following 24 hrs of morphine treatment. A-D.** Flow cytometry on ileum lamina immune cells isolated 24 hrs after implantation with placebo or 25 mg morphine pellets (n = 5). **A.** Representative flow cytometry plots of CD11b expression on B220^+^IgA^+^ B cells. **B.** Percentage of CD11b on B220^+^IgA^+^ B cells. **C**. Representative flow cytometry plots of CD11b expression on B220-IgA+ plasma cells. **D.** Percentage of CD11b on B220^-^IgA^+^ plasma cells. **E-G.** Flow cytometry on cells isolated from the Peyer’s Patches 24 hrs after implantation with placebo or 25 mg morphine pellets (n = 5). **E**. Representative flow cytometry plots of CD11b expression on IgA^+^ immune cells. **F**. Percentage of CD11b on IgA^+^ immune cells. **G**. Absolute number of IgA^+^ CD11b^+^ immune cells. Symbols represent individual mice. Mean and standard deviation are shown. Data points are pooled from 3 independent experiments. * p < 0.05 ** p < 0.01 *** p < 0.001 **** p < 0.0001 using Mann-Whitney U Test **(B, D, F,** and **G).**
