# Supplementary figures and images for: Morphine-induced intestinal microbial dysbiosis drives TLR-dependent IgA targeting of gram-positive bacteria and upregulation of CD11b and TLR2 on a sub-population of IgA+ B cells
Source: Gut Microbes. 2024 Oct 23;16(1):2417729. doi: 10.1080/19490976.2024.2417729 (PMC11508942; doi:10.1080/19490976.2024.2417729)

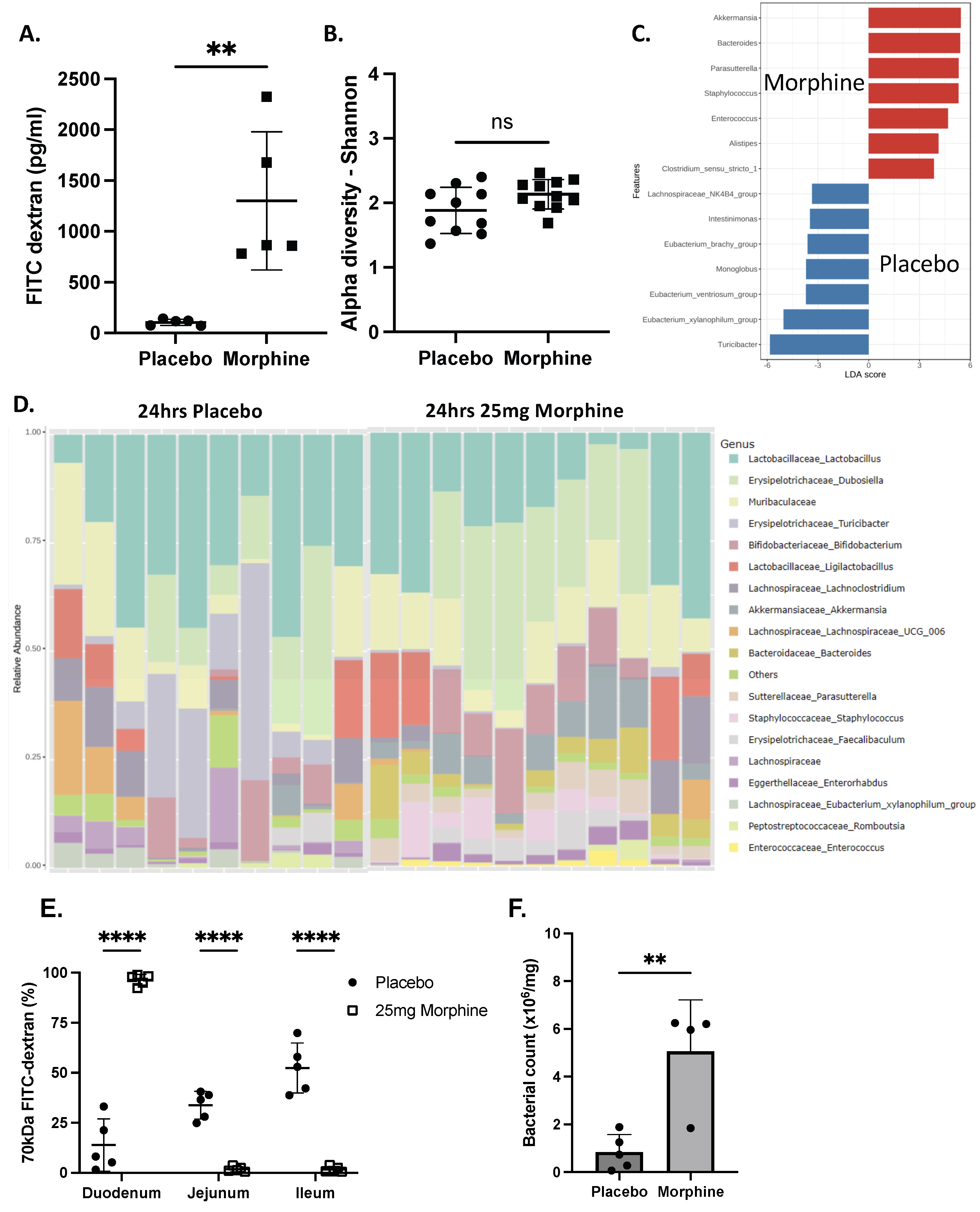

Supplement: Supplemental Material [file KGMI_A_2417729_SM8266.zip › KGMI_A_2417729/suppl_data/Figure S1.tiff]

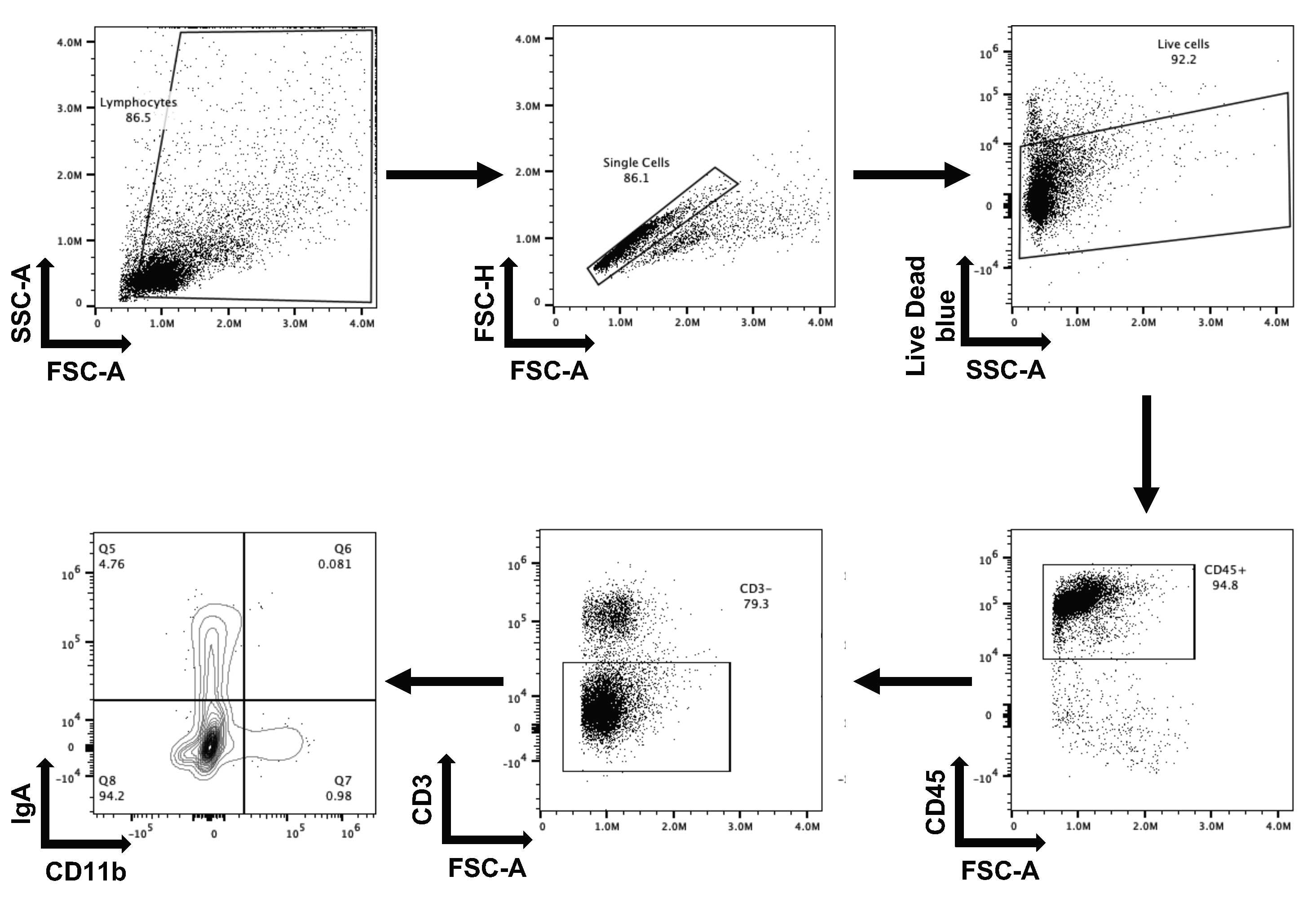

Supplement: Supplemental Material [file KGMI_A_2417729_SM8266.zip › KGMI_A_2417729/suppl_data/Figure S10.tiff]

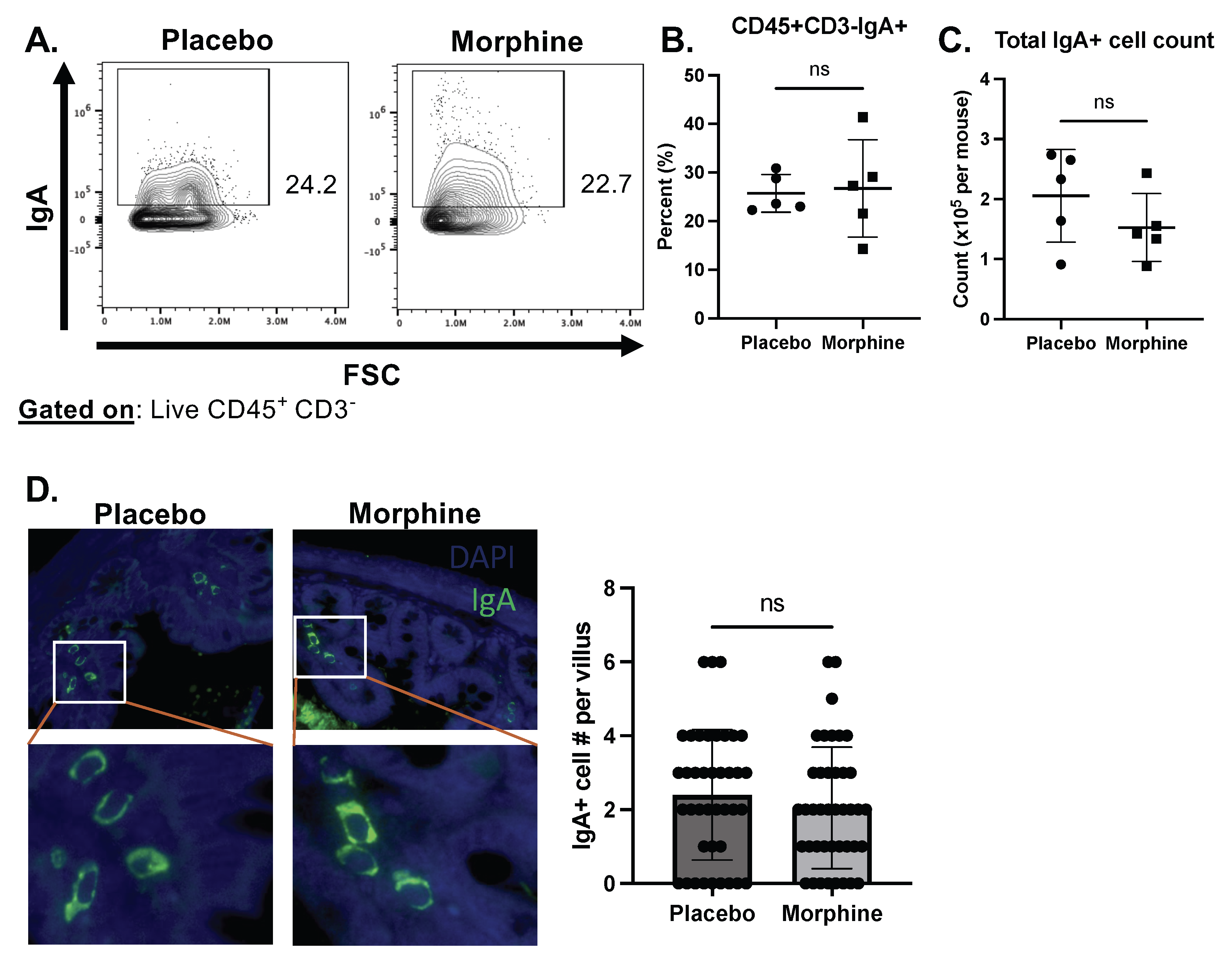

Supplement: Supplemental Material [file KGMI_A_2417729_SM8266.zip › KGMI_A_2417729/suppl_data/Figure S11.tiff]

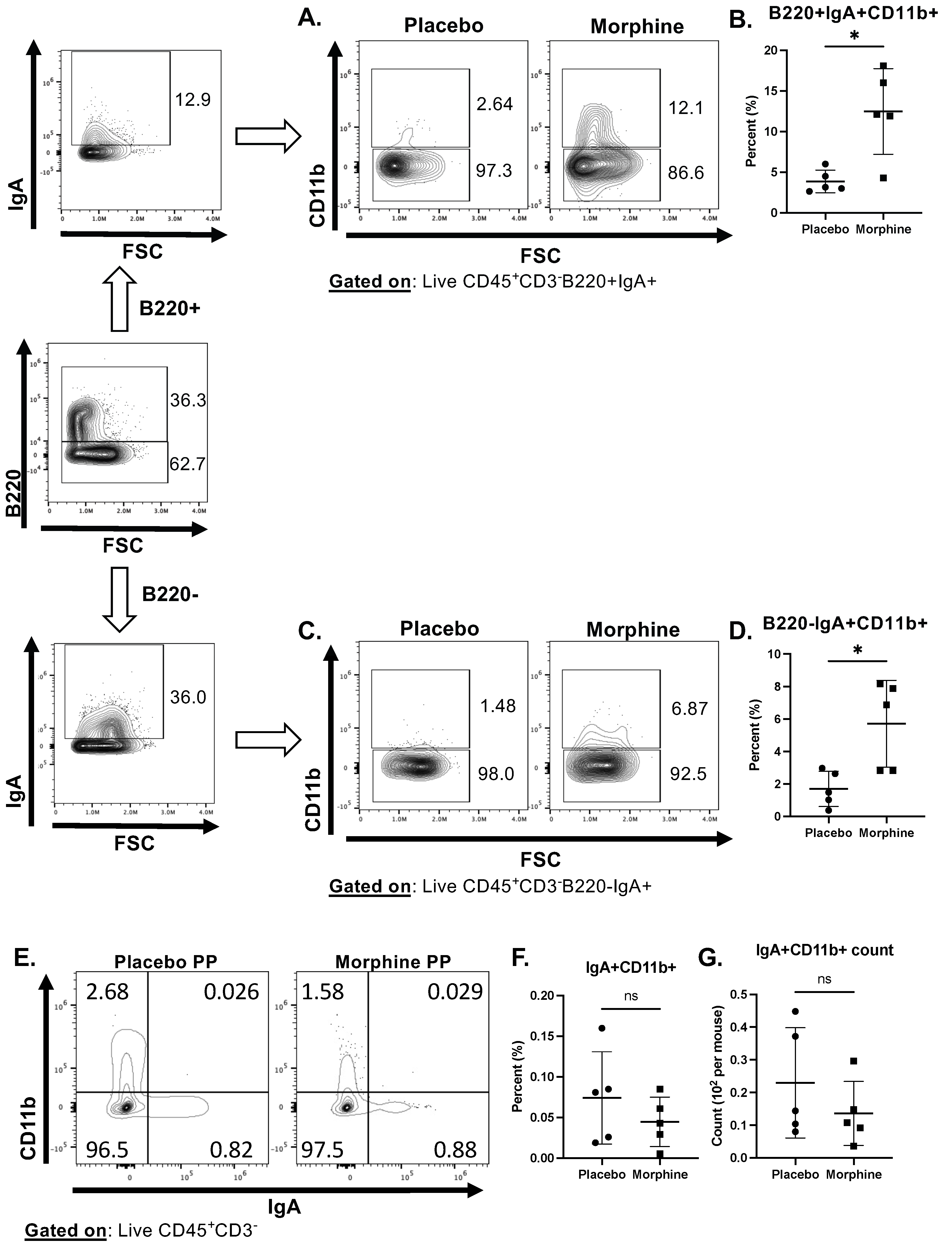

Supplement: Supplemental Material [file KGMI_A_2417729_SM8266.zip › KGMI_A_2417729/suppl_data/Figure S12.tiff]

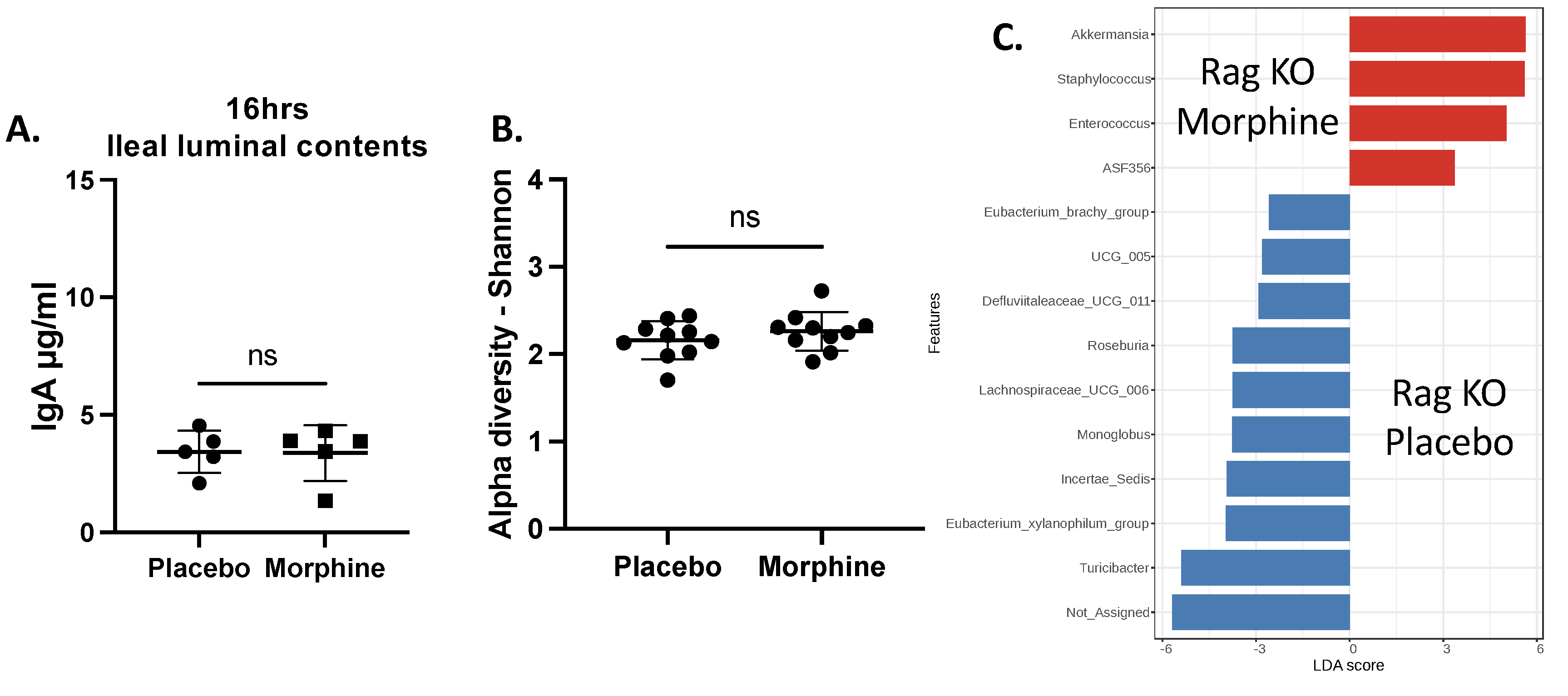

Supplement: Supplemental Material [file KGMI_A_2417729_SM8266.zip › KGMI_A_2417729/suppl_data/Figure S3.tiff]

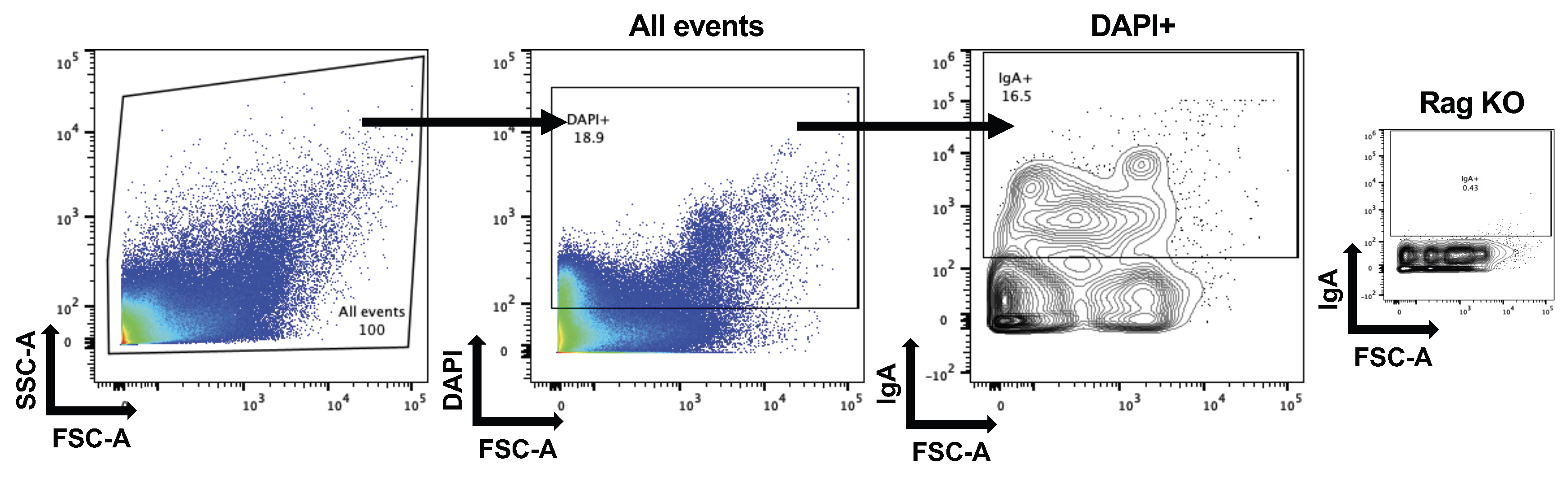

Supplement: Supplemental Material [file KGMI_A_2417729_SM8266.zip › KGMI_A_2417729/suppl_data/Figure S4.tiff]

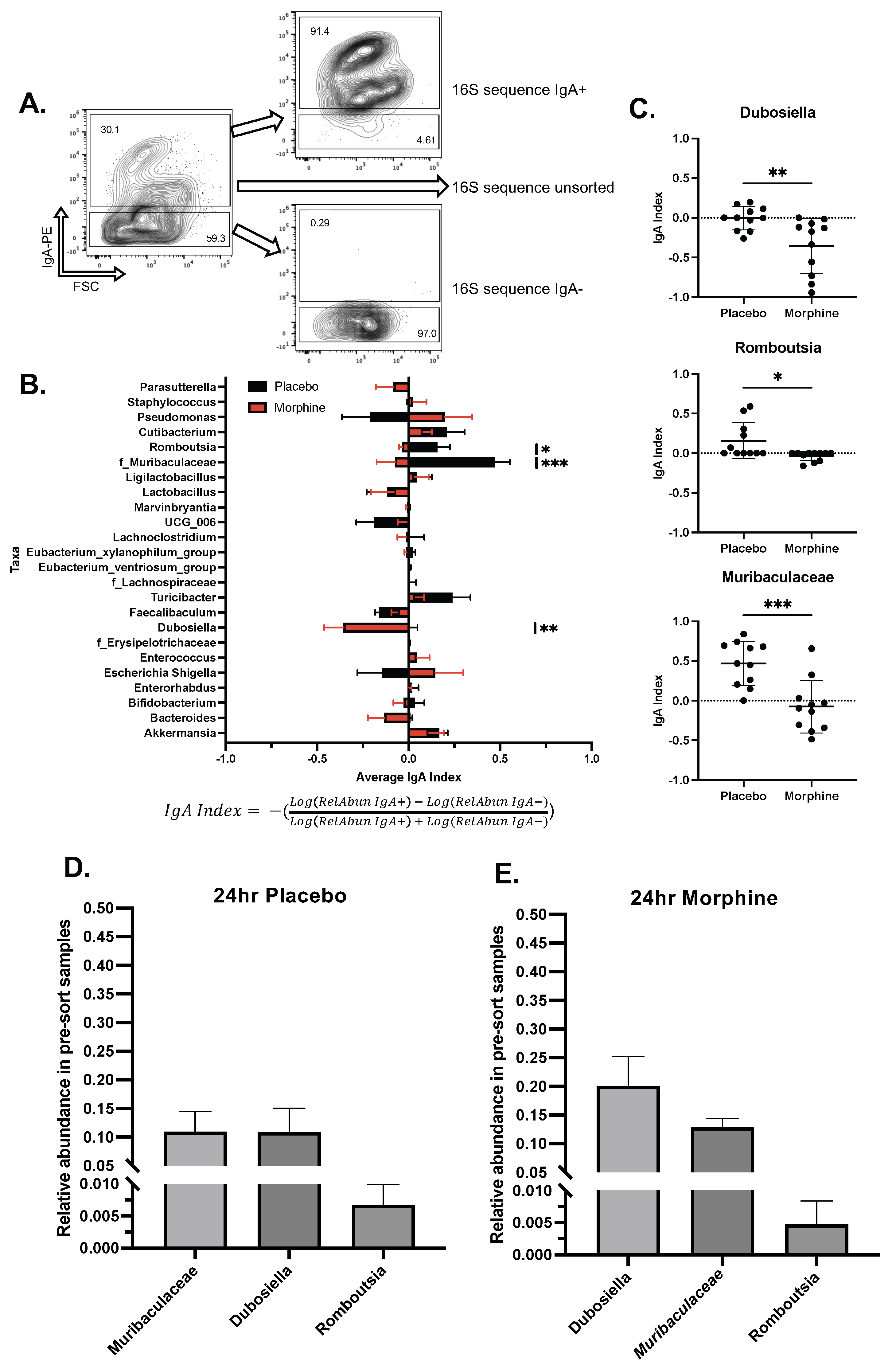

Supplement: Supplemental Material [file KGMI_A_2417729_SM8266.zip › KGMI_A_2417729/suppl_data/Figure S5.tiff]

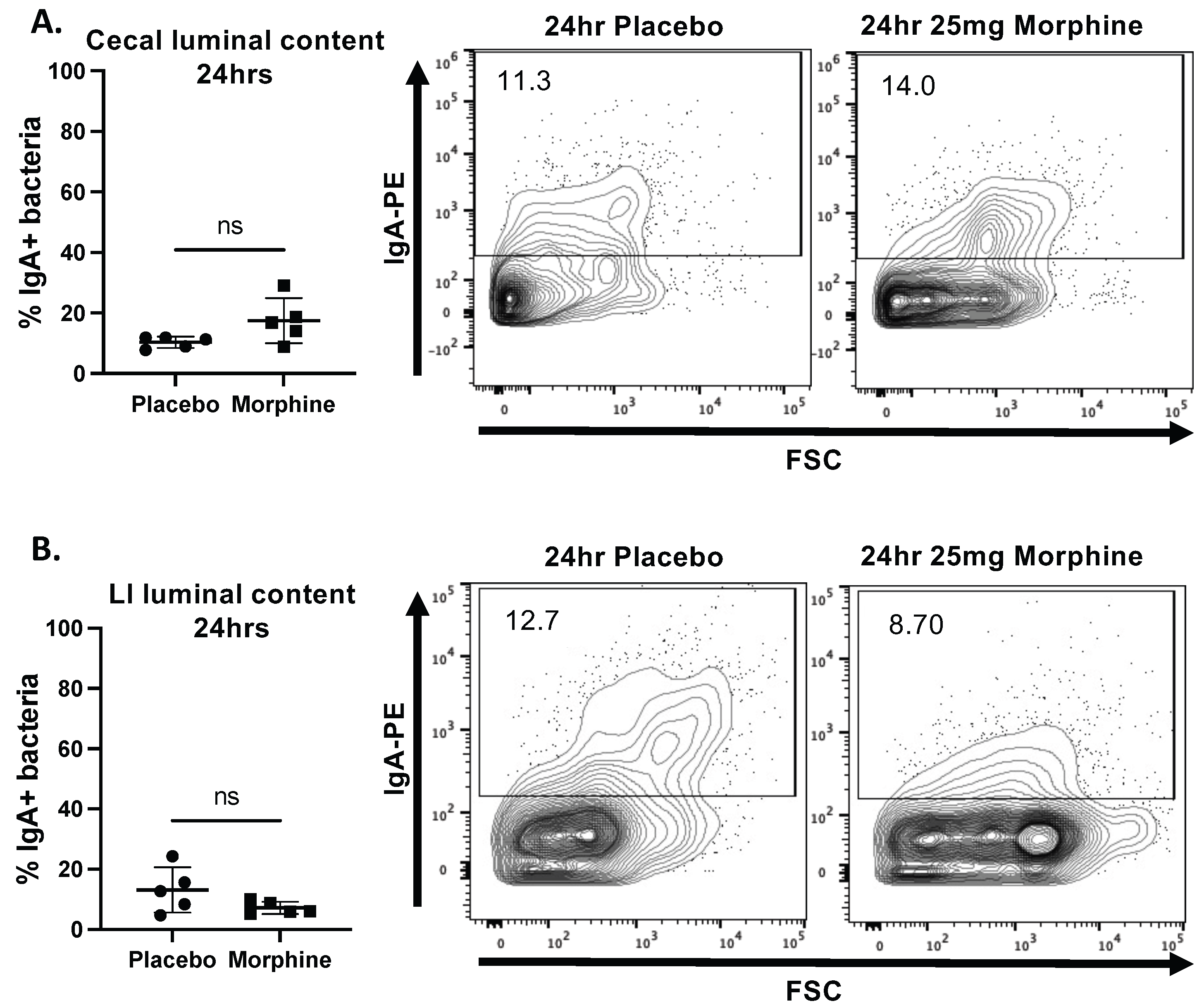

Supplement: Supplemental Material [file KGMI_A_2417729_SM8266.zip › KGMI_A_2417729/suppl_data/Figure S6.tiff]

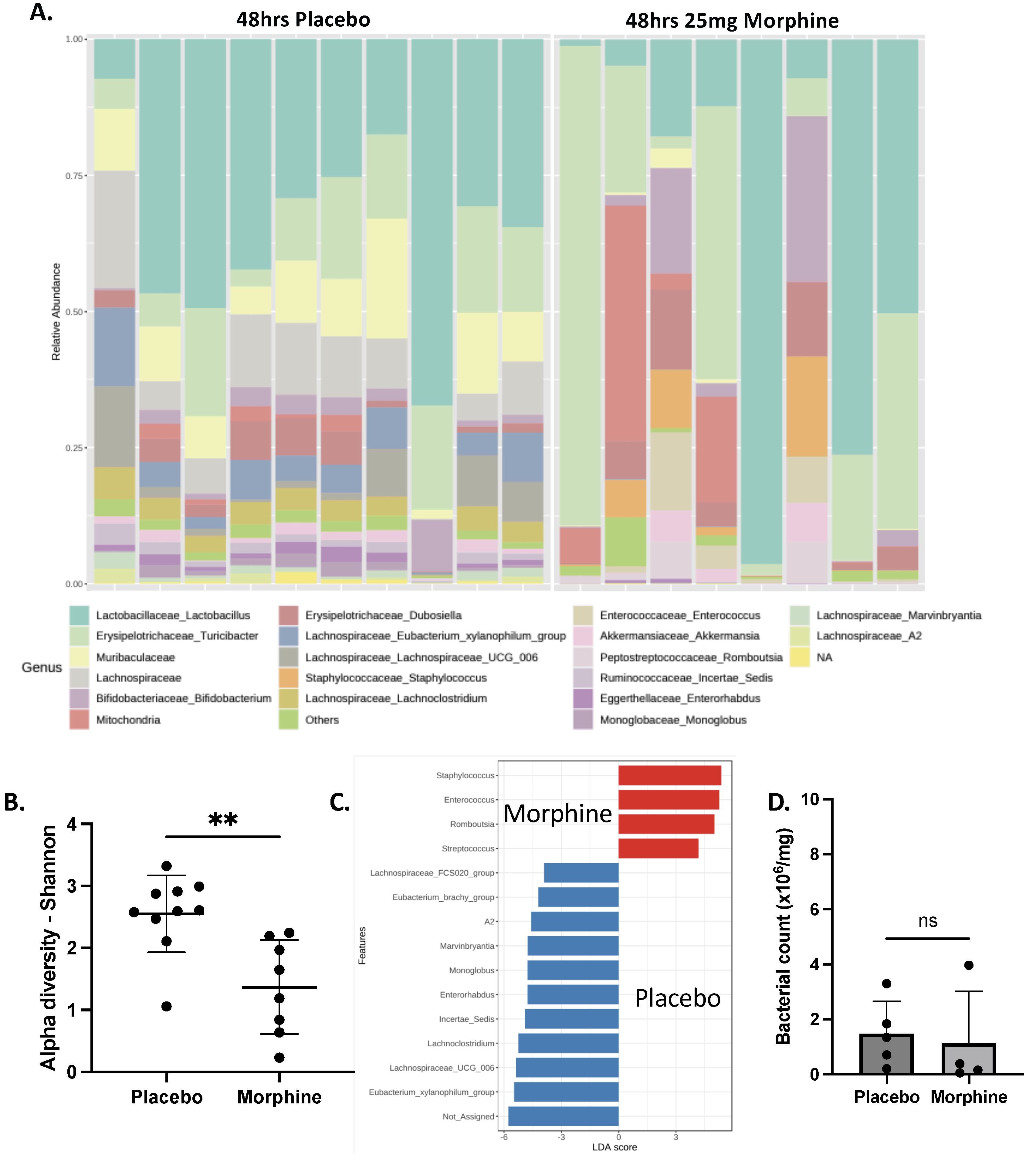

Supplement: Supplemental Material [file KGMI_A_2417729_SM8266.zip › KGMI_A_2417729/suppl_data/Figure S7.tiff]

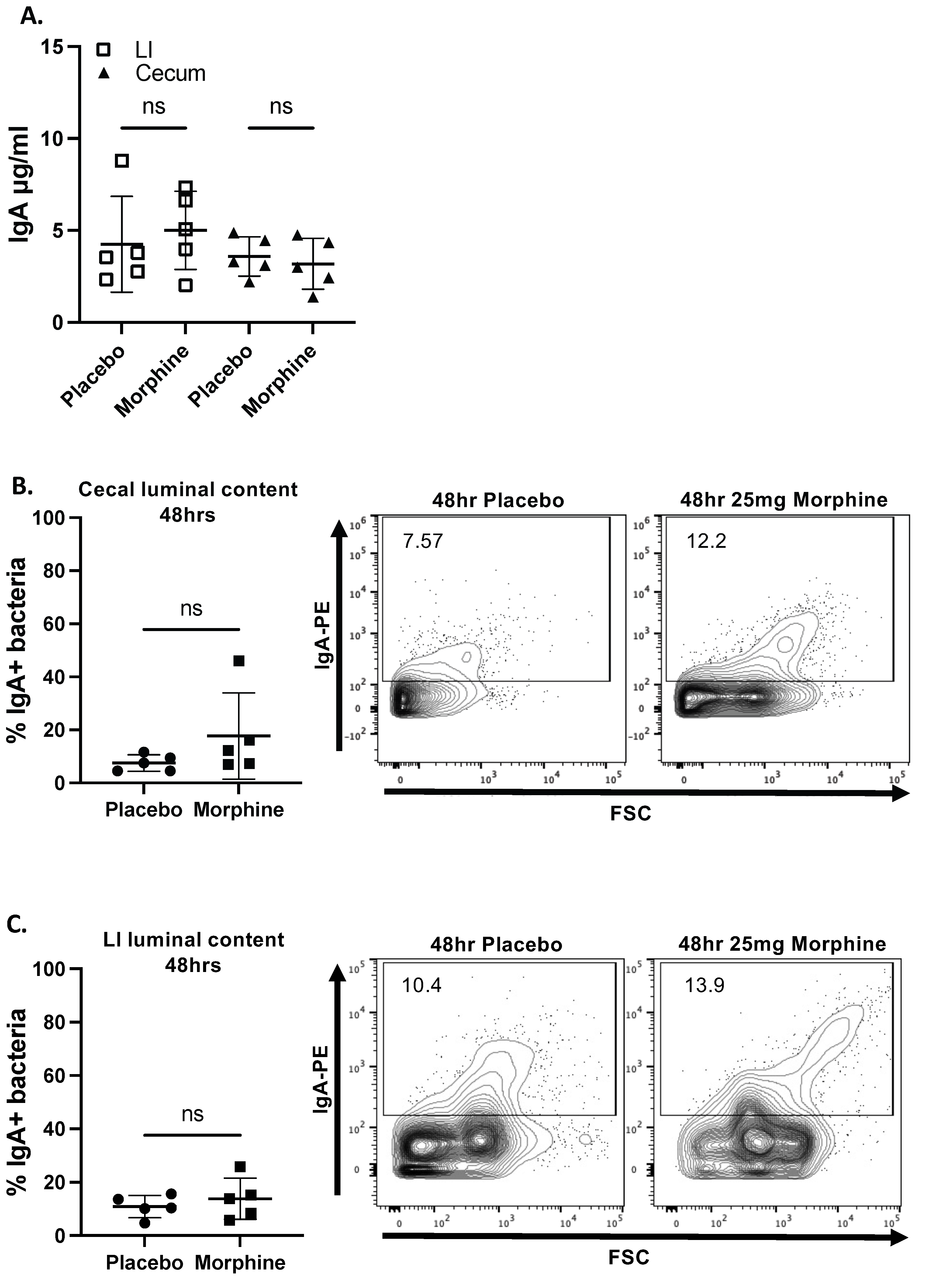

Supplement: Supplemental Material [file KGMI_A_2417729_SM8266.zip › KGMI_A_2417729/suppl_data/Figure S8.tiff]

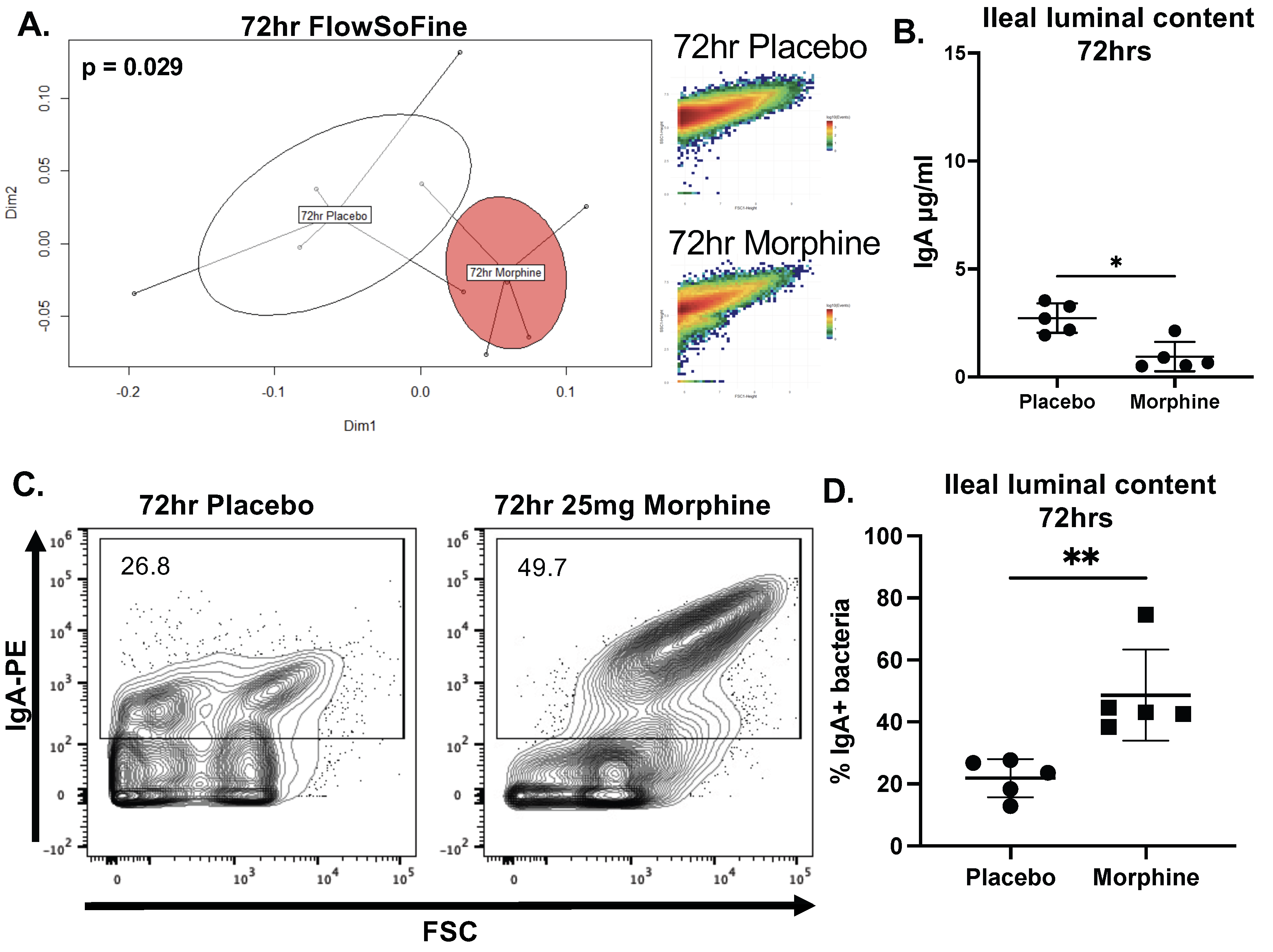

Supplement: Supplemental Material [file KGMI_A_2417729_SM8266.zip › KGMI_A_2417729/suppl_data/Figure S9.tiff]
